# Visualization of Subunit Interactions and Ternary Complexes of Protein Phosphatase 2A in Mammalian Cells

**DOI:** 10.1371/journal.pone.0116074

**Published:** 2014-12-23

**Authors:** Shu-Ting Mo, Shang-Ju Chiang, Tai-Yu Lai, Yu-Ling Cheng, Cheng-En Chung, Spencer C. H. Kuo, Kelie M. Reece, Yung-Cheng Chen, Nan-Shan Chang, Brian E. Wadzinski, Chi-Wu Chiang

**Affiliations:** 1 Institute of Molecular Medicine, College of Medicine, National Cheng Kung University, Tainan, Taiwan; 2 Institute of Basic Medical Sciences, College of Medicine, National Cheng Kung University, Tainan, Taiwan; 3 Department of Medicine, College of Medicine, National Cheng Kung University, Tainan, Taiwan; 4 Center for Infectious Disease and Signaling Research, National Cheng Kung University, Tainan, Taiwan; 5 Department of Pharmacology, Vanderbilt University Medical Center, Nashville, TN, United States of America; Faculty of Medicine, Belgium

## Abstract

Protein phosphatase 2A (PP2A) is a ubiquitous phospho-serine/threonine phosphatase that controls many diverse cellular functions. The predominant form of PP2A is a heterotrimeric holoenzyme consisting of a scaffolding A subunit, a variable regulatory B subunit, and a catalytic C subunit. The C subunit also associates with other interacting partners, such as α4, to form non-canonical PP2A complexes. We report visualization of PP2A complexes in mammalian cells. Bimolecular fluorescence complementation (BiFC) analysis of PP2A subunit interactions demonstrates that the B subunit plays a key role in directing the subcellular localization of PP2A, and confirms that the A subunit functions as a scaffold in recruiting the B and C subunits to form a heterotrimeric holoenzyme. BiFC analysis also reveals that α4 promotes formation of the AC core dimer. Furthermore, we demonstrate visualization of specific ABC holoenzymes in cells by combining BiFC and fluorescence resonance energy transfer (BiFC-FRET). Our studies not only provide direct imaging data to support previous biochemical observations on PP2A complexes, but also offer a promising approach for studying the spatiotemporal distribution of individual PP2A complexes in cells.

## Introduction

Protein phosphatase 2A (PP2A) is a major phospho-serine/threonine protein phosphatase in eukaryotic cells that regulates a variety of essential cellular events [1]. The mature PP2A holoenzyme consists of a scaffolding A subunit, a variable B regulatory subunit, and a catalytic C subunit (PP2Ac). The 36 kDa C subunit is highly conserved in eukaryotic cells, and current models suggest that prior to forming a mature PP2A holoenzyme, PP2Ac first associates with the 65 kDa A subunit to form the AC core dimer. The core dimer then associates with a third, highly variable regulatory B subunit to form a heterotrimeric holoenzyme (ABC). The diverse B regulatory subunits are thought to control the substrate specificity and subcellular localization of the PP2A holoenzyme. Four distinct B subunit families have been identified, including B (B55 or PR55) [2]–[4], B′ (B56 or PR61) [5], [6], B″ (PR72) [7] and B′′′ (PR93/PR110) [8]. The individual B subunits are differentially expressed in tissues, cells, and located in distinct subcellular compartments [1]. In the B55 subfamily, B55α, B55β1, and B55δ are primarily cytoplasmic, whereas Bβ2 is localized to mitochondria [9], and B55γ is enriched in the cytoskeletal fraction [10]. The B56 subfamily members B56α, B56β, and B56ε are mainly cytoplasmic, but B56γ1, B56γ3 and B56δ are concentrated in the nucleus [11]. These observations, together with studies of Saccharomyces cerevisiae strains lacking individual B subunit genes [12], provide support for a role of B subunit in directing the subcellular localization of the PP2A holoenzyme.

Besides association with the A and B subunits, the C subunit also forms a complex with other proteins, such as α4, which appears to be the mammalian homologue of the yeast Tap42 protein. The target of rapamycin (TOR) kinase regulates Tap42 binding with the yeast protein phosphatase catalytic subunits Pph21/22 and SIT4 [13], which are the yeast homologues of mammalian PP2A and PP6, respectively. In mammalian cells, α4 associates with the C subunit in the absence of the A and B subunits [14], [15], and participates in a wide array of cellular activities such as apoptosis [16], DNA damage response [17], and cell migration [18]. The cellular functions of α4 may be mediated via its ability to stabilize the catalytic subunits of PP2A family members (PP2Ac, PP4c, and PP6c) and prevent their degradation [17], [19], [20]. The phosphatase stabilizing role of α4 is further supported by recent structural studies, which suggest that α4 binding to PP2Ac stabilizes an inactive conformation of the phosphatase by local unfolding near the active site and steric hindrance of a ubiquitination site on PP2Ac [21]. α4 also promotes the conversion of PP2A holoenzymes to α4-PP2Ac complexes upon perturbation of the active site [21].

Most of our knowledge regarding PP2A complexes has been based on *in vitro* analyses of individual subunits or isolated complexes. However, the assembly and disassembly of PP2A oligomers may be highly dynamic and subject to regulation by various cellular cues [22]. Thus, the subcellular localization of one PP2A subunit may not necessary reflect the localization of the respective ABC holoenzyme. Although spatial and temporal changes of some PP2A subunits have been observed using immunohistochemical and fluorescent techniques, direct visualization of PP2A oligomeric complexes in cells has not been reported until now.

Several approaches have been applied to investigate protein-protein interactions, including bimolecular fluorescence complementation (BiFC) [23] and fluorescence resonance energy transfer (FRET) [24], [25]. BiFC is based on reconstituting a fluorophore by the association of two halves of a fluorescent protein when the fragments are assembled into the same macromolecular complex [23]. FRET occurs when a donor fluorophore is brought into close proximity (less than 10 nm) to an appropriate acceptor fluorophore [24], [25]. Studies of the crystal structures of PP2A complexes [26] prompted us to use BiFC [22] to visualize dimeric PP2A subunit interactions, and combined BiFC and FRET [23]–[25] to visualize ternary PP2A complexes. Our BiFC analyses not only confirmed that the A subunit functions as a scaffold for the B and C subunits, but also demonstrated that the B subunit directs the localization of PP2A holoenzymes. In addition, our BiFC studies demonstrated that α4 promotes formation of the PP2A dimeric (AC) core enzyme by stabilizing the C subunit. Moreover, for the first time, we successfully visualized two different PP2A holoenzyme complexes, Aα/B56γ3/Cα and Aα/B55β_2_/Cα, in cells by applying BiFC in conjunction with FRET (BiFC-FRET) [27].

## Materials and Methods

### Cell culture, cell lines, and transfection

NIH3T3 cells were cultured in Dulbecco's modified Eagle's medium (DMEM) supplemented with 10% bovine serum (BS). Mammalian expression plasmids were transfected into NIH3T3 cells using Lipofectamine 2000 (Invitrogen) and the manufacturer's recommended protocol. Twenty-four hours after transfection, cells were fixed or harvested for further analysis.

### DNA constructs

The construction of expression plasmids are described in S1 Text. Mammalian expression plasmids for BiFC and primers for the PCR-based cloning strategies are described in S1 and S2 Tables.

### Immunofluorescence and microscopy

Following transfection or indicated treatments, cells were washed three times with PBS, fixed by a solution containing 4% paraformaldehyde and 0.025% glutaraldehyde for 15 min, and permeabilized with 0.1% Triton-X-100 for 30 min. Cells were then blocked with 5% BSA in PBS for 1 h, and subsequently incubated with anti-HA (Cell Signaling, 2367) or anti-Myc tag (Cell Signaling, 2278) antibody for 1 h, followed by incubation with Cy3-conjugated secondary antibodies (Jackson ImmunoResearch). Cells were then stained with 4′, 6-diamidino-2-phenylindole dihydrochloride (DAPI) for 5 min, mounted, and visualized by fluorescence microscopy (Zeiss, Axio observer Z1) and confocal microscopy (Olympus, FV1000). Quantitation of subcellular distribution of immunostained proteins was carried out by both unbiased visual judgment and assessment on fluorescence intensity in both nucleus and cytoplasm using Zeiss AxioVision software.

### Co-immunoprecipitation and Western blotting

Whole cell lysates for Western analysis and immunoprecipitations were prepared in radioimmunoprecipitation assay buffer (RIPA) and isotonic immunoprecipitation buffer, respectively [28]. Immunoprecipitations were performed as previously described [29]. Briefly, whole cell lysates were incubated overnight with anti-HA (HA.11, Covance) or anti-Myc (Cell Signaling, 2278) antibodies, and the immune complexes were precipitated using protein A/G-Sepharose, followed by SDS-PAGE and Western blotting analysis using the following primary antibodies: anti-HA (Cell Signaling, 2367); anti-Myc (Cell Signaling, 2278); anti-Flag M2 (Sigma, F3165); anti-β actin (Sigma, A5441); anti-PP2A/A (Santa Cruz, sc-6112); anti-Cα (BD Transduction Laboratories, 610556). After incubation with the primary antibodies and corresponding HRP-conjugated secondary antibodies, the blots were developed by enhanced chemiluminescence.

### BiFC analysis

For analysis of interactions between A and B subunits and between A and C subunits, NIH3T3 cells were seeded at a density of 7×10^4^/well onto coverslips within 24-well plates and transiently transfected with 1 µg total DNA containing equal amounts of BiFC constructs encoding N-terminally or C-terminally YFPN-(YN-) or YFPC-(YC-) fused Aα, PP2Acα, and the B subunit in various combinations as indicated in the text. For analysis of interactions between B and C subunits, equal amounts of BiFC constructs encoding YN- or YC-fused PP2Acα and YC- or YN-fused various B subunits with or without pCA2-6MYC-Aα were co-transfected into NIH3T3 cells. For assessing the specificity of BiFC between A and B subunits in the presence of SV40 small t antigen, NIH3T3 cells were transfected with BiFC constructs encoding Aα-YC or YC-Aα, YN-fused B subunit, and pCMV5-small T_WT_, pCMV5-smallT_MUT_, or empty vector (relative amount of YC-Aα:YN-B:pCMV5-small T_WT_, -small T_MUT_, or empty vector was 1∶1∶2). For analysis of A/B complexes in cells synchronized in the early S phase, NIH3T3 cells were transfected with the indicated BiFC constructs and 24 h post-transfection, cells were treated with 10 µg/ml aphidicolin for 12 h. For analysis of interactions between PP2Ac and α4, equal amounts of BiFC constructs encoding YC-P2Acα and YN-α4_WT_ or YN-α4_MUT_ were co-transfected into NIH3T3 cells. For assessing the effect of α4 on interactions between A and C subunits, equal amounts of BiFC constructs encoding YN-Aα and PP2Acα-YC and an empty vector or a vector harboring α4_WT_, or α4_MUT_ were co-transfected into NIH3T3 cells. Twenty-four hours post-transfection, cells were washed, fixed, and visualized by fluorescence microscopy (Zeiss, Axio observer Z1) at 200× magnification and confocal microscopy (Olympus, FV1000) at 600× magnification. Quantitation of subcellular distribution of BiFC signals was carried out by both unbiased visual judgment and assessment on fluorescence intensity in both nucleus and cytoplasm using Zeiss AxioVision software.

### BiFC-FRET analysis

For visualizing PP2A AαB56γ3Cα holoenzyme complexes by BiFC-FRET analysis, NIH3T3 cells were transfected with BiFC constructs encoding YC-Aα and YN-B56γ3 and a construct for PP2Acα-CFP along with pCMV5-small T_WT_, pCMV5-small T_MUT_, or empty vector. Co-transfection of BiFC constructs encoding YC-Aα and YN-B56γ3 or transfection of PP2Acα-CFP alone was used as a control for bleed–through. For visualizing PP2A AαB55β2Cα holoenzyme complexes by BiFC-FRET analysis, NIH3T3 cells were transfected with BiFC constructs encoding YN-Aα and PP2Acα-YC and a construct for B55β2_WT_-CFP or B55β2_MUT_-CFP with or without pCMV5-small T_WT_, pCMV5-small T_MUT_, or empty vector. Twenty-four hours after transfection, cells were fixed as described earlier, and visualized and analyzed by fluorescence microscopy (Zeiss, Axio observer Z1) equipped with the FRET three-filter set (Semrock), Ex 438/24, EM 483/32, and EM 542/27. The images were sequentially acquired by CFP, YFP, FRET_CFP/YFP_ channels using image acquisition time ranging from 200 ms to 1000 ms which was kept constant for cells at random fields of different experimental groups. At least 10 cells of each set of experiments were individually measured for FRET images and corrected FRET intensity was obtained using Youvan's method [30] of AxioVision software. Each experiment was repeated at least two times.

## Results

### Immunofluorescence analysis of the subcellular distribution of individual PP2A subunits

**Figure 1:**
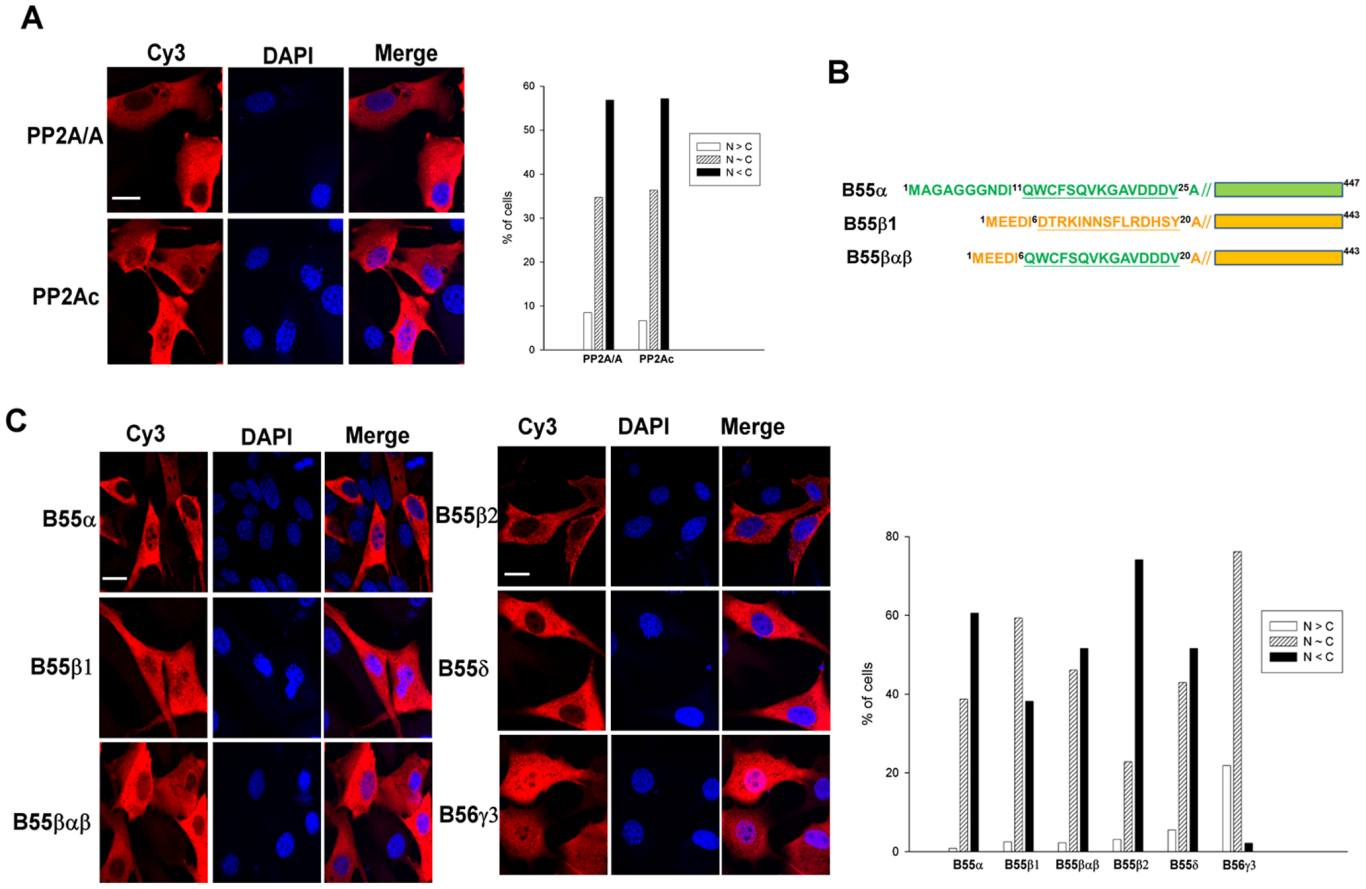
Subcellular distribution of PP2A subunits. (**A**) NIH3T3 cells were transiently transfected with pCA2-6myc-PP2A/Aα or pCMV-HA-PP2Acα-YC, and expression of the exogenous Aα and Cα subunits was assessed by indirect immunofluorescence using anti-Myc tag and anti-HA antibodies, respectively, in conjunction with Cy3-conjugated secondary antibody. (**B**) Diagrams of B55α, B55β, and the B55βαβ chimera mutant are shown. (**C**) NIH3T3 cells were transiently transfected with pcDNA3.1/Zeo(+)-B55α-HA, pcDNA3.1/Zeo(+)-B55β-HA, pcDNA3.1/Zeo(+)-B55βαβ-HA, pcDNA3.1/Zeo(+)-B55β2-HA, pcDNA3.1/Zeo(+)- B55δ-HA, or pcDNA3.1/Zeo(+)-B56γ3-HA. Expression of various exogenous B isoforms was assessed by indirect immunofluorescence using the anti-HA antibody and Cy3-conjugated secondary antibody. DAPI was applied for staining of nuclei. Scale bars: 20 µm. Cells with different distribution patterns were scored as follows: predominantly nuclear (N>C), homogenously distributed in both nucleus and cytoplasm (N∼C), and predominantly cytoplasmic (N<C). Quantified data from one of at least two independent experiments with similar results are shown. At least 200 cells were counted for each group.

We performed indirect immunofluorescence analyses to visualize the localization of Myc-tagged PP2A/Aα and HA-tagged PP2Acα in NIH3T3 cells. As shown in Fig. 1A, nearly 58% of the cells displayed a predominantly cytoplasmic expression pattern for both Myc-tagged PP2A/Aα and HA-tagged PP2Acα subunits, and approximately 35% of the cells displayed ubiquitous expression of these PP2A subunits. We also examined the localization pattern of various HA-tagged B subunits including B55α, B55β1, B55β2, B55δ, and B56γ3 (Fig. 1C). B55α and B55δ, which share the highest sequence homology among B55 family members [31], showed mainly cytoplasmic distribution. B55β1 was ubiquitously expressed in entire cells or predominantly cytoplasmic, but B55β2 was mainly cytoplasmic. Consistent with a previous investigation that showed B55β2, but not B55β1, is located to mitochondria [9], we found that the distribution of B55β2 was more punctate, which is a hallmark of mitochondrial localization. B56γ3, which is a member of the B56 family of the regulatory subunits, was ubiquitously distributed in most of the cells and was highly enriched in the nucleus of a subset of cells. We also examined the localization of B55βαβ, which is a chimera of B55β1 with amino acid residues 6 to 20 replaced by amino acid residues 11 to 25 of B55α (Fig. 1B). As shown in Fig. 1C, the cytoplasmic distribution of B55βαβ was increased relative to that of wildtype B55β1, and more closely resembled the distribution pattern of B55α. This finding suggests that the very diverse region at the N-terminus of B55α and B55β1 plays a key role in regulating the subcellular targeting of these highly similar subunits. Together, in agreement with prior studies [9]–[11], [32], our immunofluorescence analyses of individual B subunits indicate that they display unique subcellular localization patterns.

### BiFC analysis of subunit interactions

**Figure 2:**
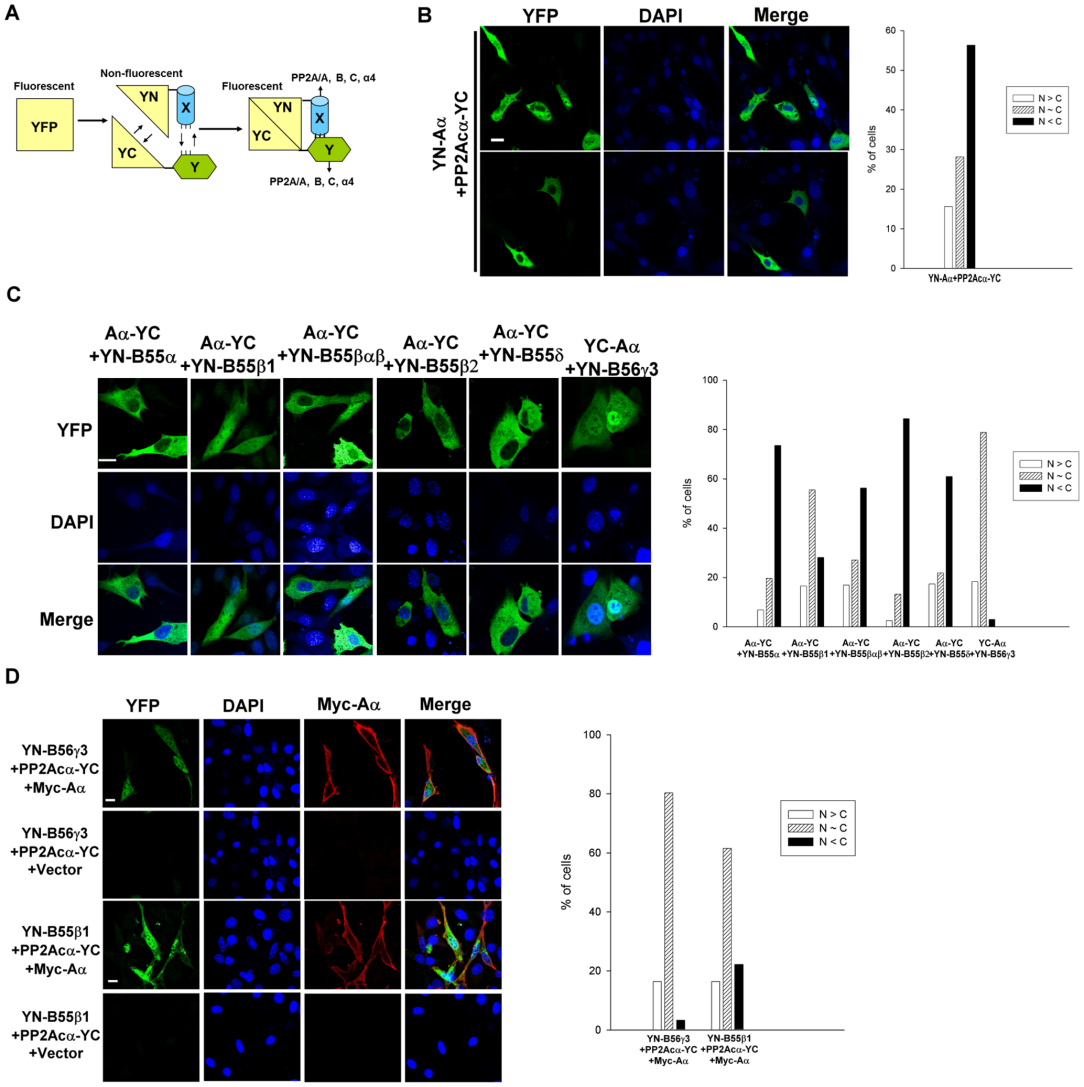
BiFC analysis enables visualization of association between two subunits of PP2A in cells. (**A**) Design of BiFC analysis of dimeric interactions between PP2A subunits is shown. Fluorescence is regained when reconstitution of YFP from two fragments of YFP takes place due to an interaction between PP2A subunits fused to the fragments. (**B**) Equal amounts of BiFC expression constructs encoding YN-Aα and PP2Acα-YC were co-transfected into NIH3T3 cells. YFP signals due to BiFC of YN-Aα and PP2Acα-YC were measured by fluorescence microscopy. (**C**) Equal amounts of BiFC expression constructs encoding Aα-YC and YN-B55α, YN-B55β1, YN-B55β2, YN-B55βαβ, or YN-B55δ, or constructs encoding YC-Aα and YN-B56γ3 were transfected into NIH3T3 cells. YFP signals due to BiFC of Aα-YC and YN-B were measured by fluorescence microscopy. (**D**) Equal amounts of BiFC expression constructs encoding PP2Acα-YC and YN-B55β1 or YN-B56γ3 with or without equal amounts of pCA2-6myc-PP2A/Aα were co-transfected into NIH3T3 cells, and 24 h after transfection, YFP signals due to BiFC of PP2Acα-YC and YN-B55β1 or YN-B56γ3 were measured by direct fluorescence microscopy and expression of 6myc-PP2A/Aα was confirmed by indirect immunofluorescence using anti-Myc tag antibody and Cy3-conjugated secondary antibody. DAPI was applied for staining of nuclei. Scale bars: 20 µm. Graphs show quantitative analysis of distribution of BiFC signals in cells from one of at least two independent experiments with similar results, and at least 100 cells were assessed from several random fields. Cells with different distribution patterns of BiFC signals were scored as described earlier.

To investigate the interaction of canonical PP2A subunits (e.g., A and C, A and B, and B and C), we exploited BiFC (Fig. 2A). Mammalian expression plasmids encoding Aα, PP2Acα, B55α, B55β1, B55β2, B55βαβ, B55δ, or B56γ3 fused to an N-terminal fragment of yellow fluorescent protein (YN) or to a C-terminal fragment of yellow fluorescent protein (YC), at either the N terminus or C terminus of the PP2A subunit, were generated and transfected into NIH3T3 cells. The BiFC efficiency of paired YN- and YC-fused PP2A subunits was assessed by fluorescence microscopy. Our analysis of various pairs of YN- or YC-fused Aα and PP2Acα subunits revealed that the highest BiFC signals were generated from the combination of YN-Aα and PP2Acα-YC (Fig. 2B and S1 Fig.). In agreement with the results of indirect immunofluorescence of Aα and PP2Acα (Fig. 1A), the BiFC signals of YN-Aα/PP2Acα-YC complexes were mainly cytoplasmic in most cells; however, some cells exhibited fairly ubiquitous or nuclear-enriched localization of these complexes (Fig. 2B).

Next, we investigated the interactions between A and B subunits using BiFC. Our analyses of various pairs of YN- or YC-fused Aα and B55α, B55β1, or B55βαβ subunits revealed that the highest BiFC signals were obtained from the combination of Aα-YC or YC-Aα and YN-B for all three tested B subunits (S2–S4 Figs.). Our BiFC studies revealed that Aα-YC/YN-B55α, Aα-YC/YN-B55β2, Aα-YC/YN-B55βαβ, and Aα-YC/YN-B55δ complexes were mainly cytoplasmic, and to a much lesser extent, in a ubiquitous or in a nuclear-enriched manner (Fig. 2C). However, BiFC signals of Aα-YC/YN-B55β1 and YC-Aα/YN-B56γ3 complexes were mainly ubiquitous, but some cells exhibited highly nuclear-enriched BiFC signals for YC-Aα/YN-B56γ3 complexes (Fig. 2C). Moreover, consistent with the results of indirect immunofluorescence analysis of individual B subunits (Fig. 1C), Aα-YC/YN-B55βαβ complexes displayed a more predominant cytoplasmic distribution than Aα-YC/YN-B55β1 complexes, which were ubiquitously distributed. This observation confirms that the divergent N-terminal regions of B55α and B55β play an important role in regulating the subcellular targeting of two very similar subunits. Co-immunoprecipitation experiments not only verified that Aα-YC or YC-Aα can interact with the various YN-B subunits (S5 Fig.), but also demonstrated association of the endogenous PP2A/A and PP2Ac subunits with the YN-B subunits (S5 Fig.). These data indicate that the BiFC signals formed between fluorescent protein fragments fused with A and B subunits most likely represent the trimeric ABC holoenzyme complex.

We extended our BiFC analyses to explore interactions between the B and C subunit. We chose the B55β, B55δ and B56γ3 subunits for investigation because they showed higher BiFC signals than the other B subunits (Fig. 2C). No appreciable BiFC signals could be detected following expression of different pairs of YN- or YC-fused PP2Acα and YN- or YC-fused B55β, B55δ and B56γ3 subunits (S6–S8 Figs.). However, marked BiFC signals were detected when Aα was co-expressed with PP2Acα-YC and either YN-B56γ3 or YN-B55β1 (Fig. 2D and S6–S7 Figs.). These findings verify that the A subunit functions as a scaffold for the B and C subunits. The BiFC signals for PP2Acα/B56γ3 and PP2Acα/B55β1 in the presence of Aα also showed distribution patterns similar to those observed for Aα/B56γ3 and Aα/B55β1 complexes, respectively (compare Fig. 2C, D). Although B55δ shares high similarity with B55β, no obvious BiFC signals were detected in all combinations of YN- or YC-fused PP2Acα and B55δ regardless of whether or not Aα was co-expressed (S8 Fig.). We also found no significant differences between the expression levels of YN-B55β1 and YN-B55δ, and association of PP2Acα-YC with YN-B55β1 and YN-B55δ (S9 Fig.).

**Figure 3:**
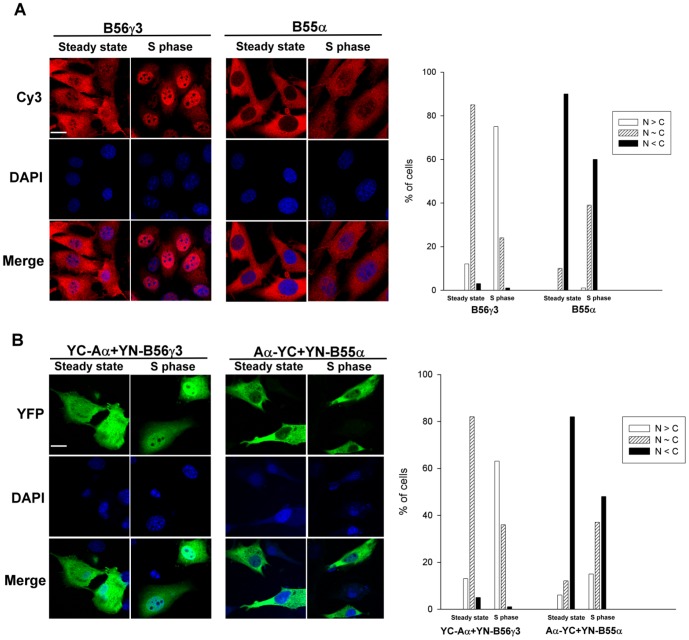
BiFC analysis confirms that B56γ3, but not B55α, promotes PP2A/Aα accumulation in the nucleus in early S phase. (**A**) NIH3T3 cells stably expressing HA-tagged B56γ3 or B55α were synchronized at the early S phase by double thymidine block treatment [48] followed by released in the regular medium for 3 h as described before [29]. Expression of B56γ3 or B55α was assessed by indirect immunofluorescence using anti-HA antibody in conjunction with Cy3-conjugated secondary antibody. (**B**) Equal amounts of BiFC expression constructs encoding YC-Aα and YN-B56γ3 or equal amounts of BiFC expression constructs encoding Aα-YC and YN-B55α were co-transfected into NIH3T3 cells. Twenty-four hour after transfection, cells were either treated with 10 µg/ml aphidicolin or left untreated for 18 h and subsequently grown in fresh medium without aphidicolin treatment for 3 h, followed by direct fluorescence microscopy for imaging YFP signals due to BiFC of YC-Aα and YN-B56γ3 or BiFC of Aα-YC and YN-B55α. DAPI was applied for staining of nuclei. Scale bars, 20 µm. Graphs show quantitative analysis of distribution of BiFC signals in cells from one of at least two independent experiments with similar results, and at least 100 cells were assessed from several random fields. Cells with different distribution patterns of BiFC signals were scored as described earlier.

Since we previously showed that B56γ3 is enriched in the nucleus at the G1 to S transition and at early S phase [29], we applied BiFC analysis to determine if complexes of Aα/B56γ3 were regulated in a similar fashion. Consistent with our prior study [29], immunofluorescence analysis revealed that, in comparison to asynchronous cells, B56γ3 became highly enriched in the nucleus following cell synchronization at early S phase, whereas B55α showed only a moderate increase in the nuclear distribution when cells were synchronized at early S phase (Fig. 3A). Likewise, we found that BiFC complexes of Aα/B56γ3 were homogenously distributed in entire cells, but became highly enriched in the nucleus in cells synchronized at early S phase (Fig. 3B). In contrast, only a moderate increase in nuclear distribution of Aα/B55α BiFC complexes was found at early S phase as compared to that at steady state (Fig. 3B). Collectively, these results indicate that BiFC complexes of A and B subunits behave similarly to individual B subunits (Table 1). Together, these findings confirm that the A subunit functions as a scaffold for the B and C subunits, and demonstrate that the B subunit dictates the subcellular localization of canonical PP2A holoenzymes.

**Table 1:** Subcellular distribution of different subunits and BiFC complexes of PP2A.

Subunit	Location Major/secondary	Subunit	Location Major/secondary	BiFC complex	Location Major/secondary
		B55α	Cytoplasmic/Ubiquitous	Aα/B55α	Cytoplasmic/Ubiquitous
		B55β1	Ubiquitous/Cytoplasmic	Aα/B55β1	Ubiquitous/Cytoplasmic
Aα	Cytoplasmic/Ubiquitous	B55βαβ	Either Cytoplasmic or Ubiquitous	Aα/B55βαβ	Cytoplasmic/Ubiquitous
		B55β2	Cytoplasmic Punctate/Ubiquitous	Aα/B55β2	Cytoplasmic/Ubiquitous
		B55δ	Cytoplasmic/Ubiquitous	Aα/B55δ	Cytoplasmic/Ubiquitous
		B56γ3	Ubiquitous/Nuclear- enriched	Aα/B56γ3	Ubiquitous/Nuclear- enriched
		Cα	Cytoplasmic/Ubiquitous	Aα/Cα	Cytoplasmic/Ubiquitous
Cα	Cytoplasmic/Ubiquitous	B55β1	Ubiquitous/Cytoplasmic	Cα/B55β1	Ubiquitous/Cytoplasmic
		B56γ3	Ubiquitous/Nuclear- enriched	Cα/B56γ3	Ubiquitous/Nuclear- enriched

### SV40 small t antigen disrupts BiFC between Aα and various B subunits

**Figure 4:**
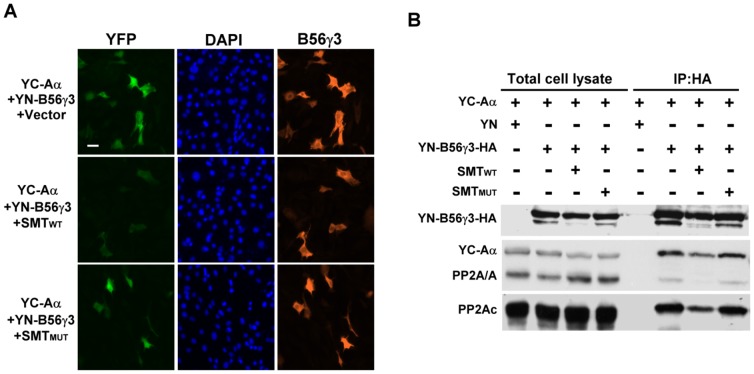
BiFC between YC-Aα and YN-B56γ3 is disrupted by SV40 SMT. (**A**) BiFC expression constructs encoding YC-Aα and YN-B56γ3 in the presence of pCMV5-SMT_WT_, -SMT_MUT_, or empty vector were transfected into NIH3T3 cells, and 24 h after transfection, YFP signals due to BiFC of YC-Aα and YN-B56γ3 were measured by fluorescence microscopy. Expression of HA-tagged YN-B56γ3 was confirmed using anti-HA antibody as described earlier. (**B**) BiFC expression constructs encoding YC-Aα and YN-B56γ3 in the presence of pCMV5 SMT_WT_, -SMT_MUT_, or empty vector were transfected into NIH3T3 cells. Cell lysates were collected 24 h post-transfection, and immunoprecipitation was performed using anti-HA-Sepharose. The cell lysates and anti-HA immunocomplexes were then analyzed by SDS-PAGE and Western blotting using indicated antibodies.

Since SV40 small t antigen (SMT) binds to the A subunit and displaces B subunits from PP2A heterotrimeric holoenzymes [33], we investigated whether SMT can disrupt BiFC complexes formed between A and B subunits. YC-Aα and YN-B56γ3 were co-expressed with wildtype SV40 small t antigen (SMT_WT_) or a mutant form of SV40 small t antigen (SMT_MUT_) defective in binding the A subunit [34]. Expression of SMT_WT_, but not the SMT_MUT_, abolished the BiFC signal generated from co-expression of YC-Aα and YN-B56γ3 (Fig. 4A). SMT_WT_ also significantly attenuated the BiFC signals in cells expressing Aα-YC and YN-B55α, YN-B55β1, YN-B55β2, YN-B55βαβ, or YN-B55δ, whereas SMT_MUT_ did not affect the BiFC complexes (S10 Fig.). Results from co-immunoprecipitation experiments confirmed that SMT_WT_, but not SMT_MUT_, significantly disrupted the interaction of YN-B56γ3 with Aα-YC as well as with endogenous Aα and Cα subunits (Fig. 4B). These findings indicate that the BiFC signals detected following co-expression of Aα-YC and various YN-fused B subunits are indeed due to specific protein-protein interactions and not the result of spontaneous interactions between two non-fluorescent fragments of a split YFP.

### BiFC analysis of PP2Ac/α4 complexes

**Figure 5:**
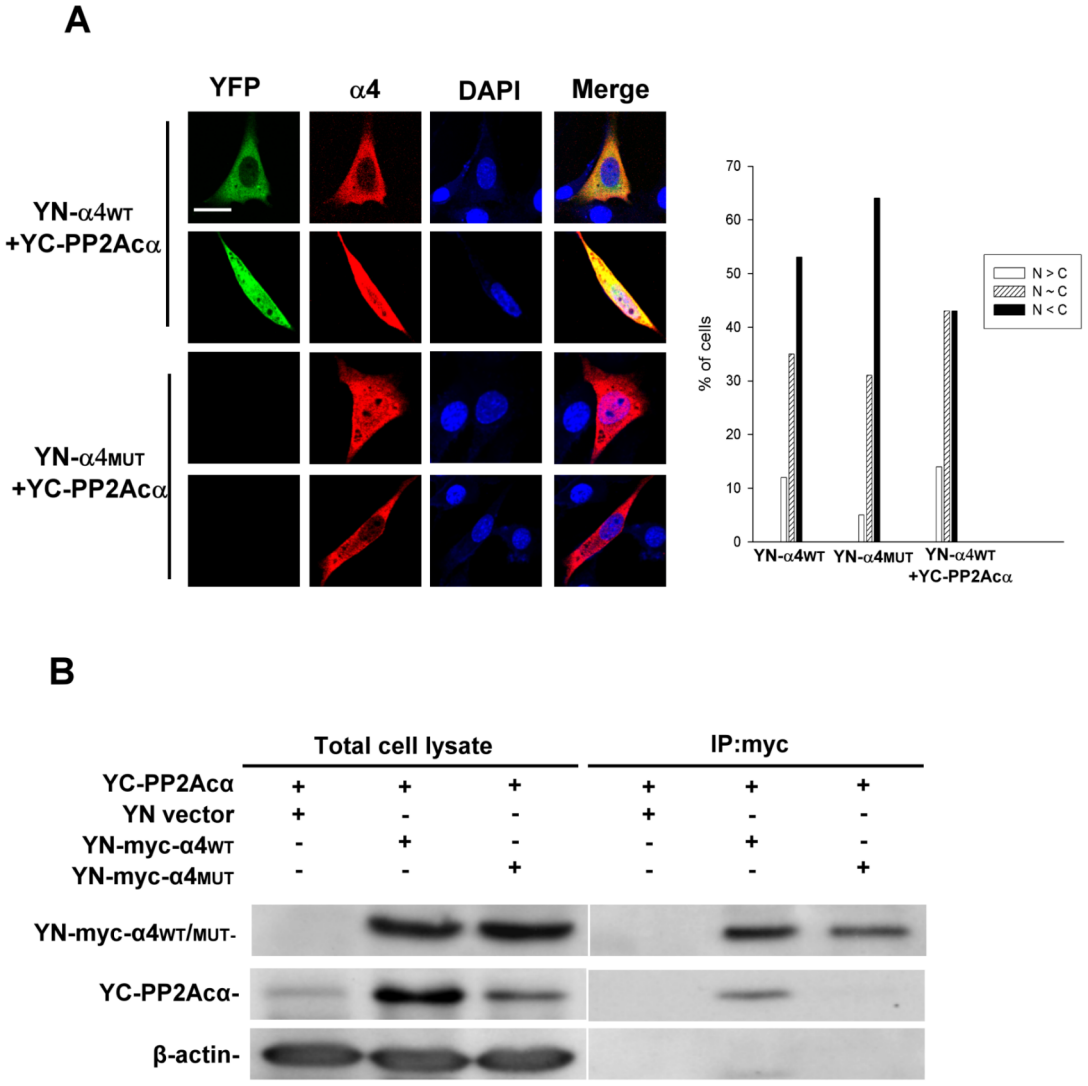
Visualization of the α4/PP2Ac complex by BiFC. (**A**) BiFC expression constructs encoding YN-α4_WT_ or YN-α4_MUT_ and YC-PP2Acα were transfected into NIH3T3 cells. YFP signals due to BiFC of YN-α4_WT_ or BiFC of YN-α4_MUT_ and YC-PP2Acα were measured by fluorescence microscopy. Expression of Myc-tagged YN-α4_WT_ or YN-α4_MUT_ was confirmed using anti-Myc antibody in conjunction with Cy3-conjugated secondary antibody by indirect immunofluorescence microscopy. DAPI was applied for staining of nuclei. Scale bars: 20 µm. Cells with different distribution patterns were scored as described earlier. Quantified data from one of at least two independent experiments with similar results are shown. At least 150 cells were counted for each group. (**B**) BiFC expression constructs encoding YN-α4_WT_ or YN-α4_MUT_ and YC-PP2Acα were transfected into NIH3T3 cells. Cell lysates were collected 24 h post-transfection and immunoprecipitations were performed using anti-Myc tag antibody. The cell lysates and anti-Myc tag immunocomplexes were then analyzed by SDS-PAGE and Western blotting using the indicated antibodies.

Given that BiFC can be utilized to detect dimeric interactions of canonical PP2A subunits (Fig. 2), we applied it to investigate a non-canonical PP2Ac complex, namely the PP2Ac/α4 complex. Indirect immunofluorescence revealed that both YN-tagged wildtype alpha4 (α4_WT_) and a mutant alpha4 defective in PP2Ac binding (α4_MUT_) [35], [36] were distributed in a primarily cytoplasmic manner and, to a lesser extent, in a ubiquitous manner (Fig. 5A, α4 panels). Co-expression of YN-α4_WT_ and YC-PP2Acα resulted in significant BiFC signals, whereas no apparent BiFC signals were detected when the YN-α4_MUT_ was co-expressed with YC-PP2Acα (Fig. 5A, YFP panels). Like the expression pattern of PP2Ac (Fig. 1) and α4 observed by immunofluorescence (Figs. 1 and 5A), the subcellular distribution of YC-PP2Acα/YN-α4_WT_ complexes was either ubiquitous or mainly cytoplasmic, and to a much lesser extent, nuclear-enriched (Fig. 5A, graph). To verify the YN-α4/YC-PP2Acα BiFC data, we performed co-immunoprecipitation experiments. As shown in Figure 5B, YN-α4_WT_, but not YN-α4_MUT_, associated with YC-PP2Acα.

**Figure 6:**
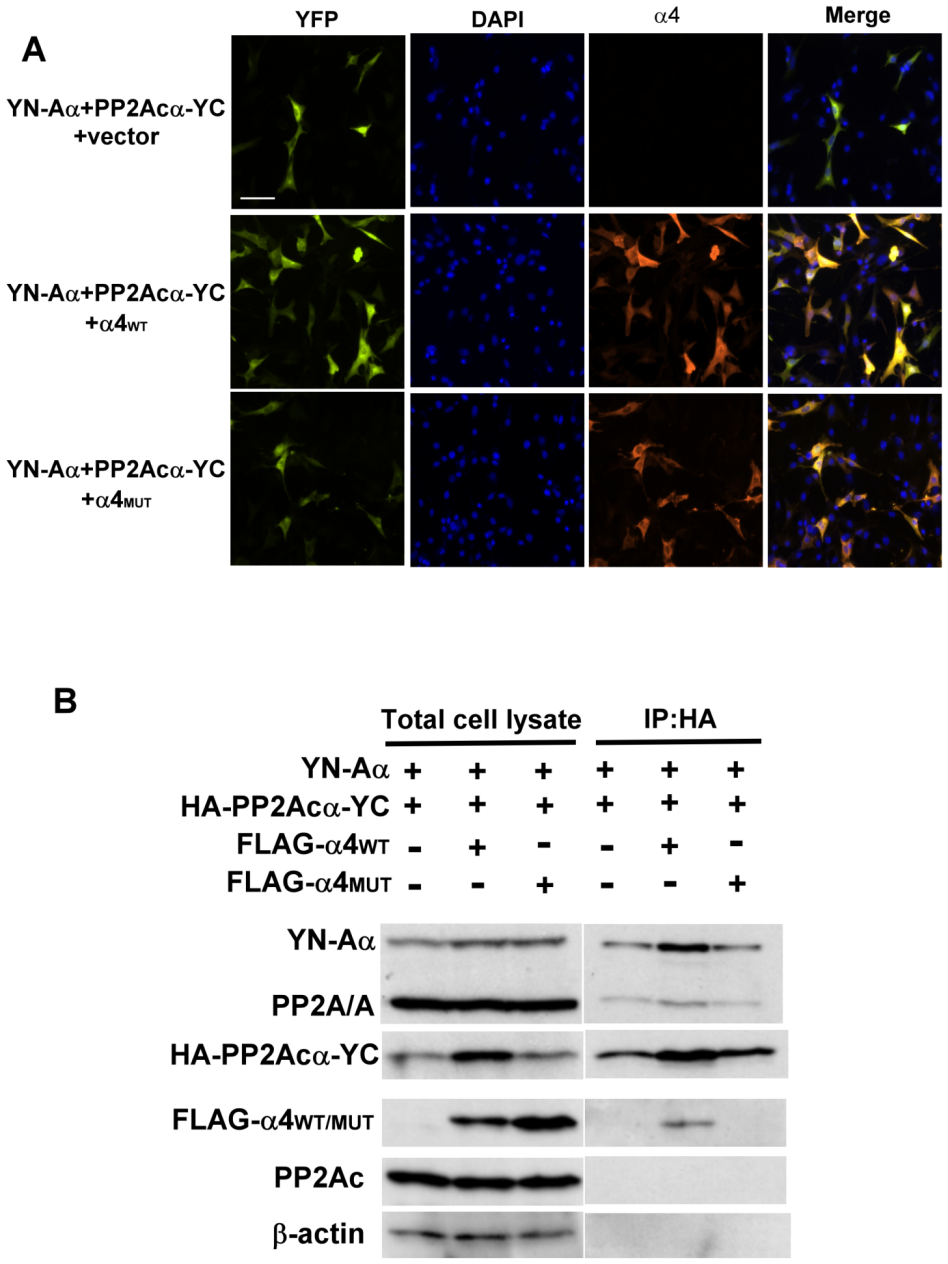
α4 facilitates formation of PP2A AC core enzyme. (**A**) Equal amounts of BiFC expression constructs encoding YN-Aα and PP2Acα-YC in the presence of equal amounts of pcDNA5/To-Flag-α4_WT_, pcDNA5/To-Flag-α4_MUT_, or empty vector were transfected into NIH3T3 cells. YFP signals due to BiFC of YN-Aα and PP2Acα-YC were measured by fluorescence microscopy. Expression of Flag-α4_WT_ or Flag-α4_MUT_ was verified using anti-FLAG antibody and Cy3-conjugated secondary antibody. DAPI was applied for staining of nuclei. Scale bars, 20 µm. (**B**) BiFC expression constructs encoding YN-Aα and HA-PP2Acα-YC and pcDNA5/To-Flag-α4_WT_, pcDNA5/To-Flag-α4_MUT_, or empty vector were transfected into NIH3T3 cells. Cell lysates were collected 24 h post-transfection, and immunoprecipitations were performed using anti-HA Sepharose. The cell lysates and anti-HA immunocomplexes were then analyzed by SDS-PAGE and Western blotting using the indicated antibodies.

The α4 and A subunits directly associate with C subunit in a mutually exclusive fashion and α4 appears to compete with the A subunit for binding the C subunit [13], [14]. To test the competition hypothesis using BiFC, we co-expressed YN-Aα and PP2Acα-YC together with or without α4. Surprisingly, we found that co-expression of α4_WT_ resulted in increased BiFC signals of YN-Aα/PP2Acα-YC complexes (Fig. 6A). The increased YN-Aα/PP2Acα-YC BiFC signals were not observed when the PP2Ac binding-defective mutant of α4 was introduced into the cells (Fig. 6A). The α4-induced increases in YN-Aα/PP2Acα-YC BiFC signals were further verified by co-immunoprecipitation and Western blotting analysis. These experiments revealed that co-expression of α4_WT_, but not α4_MUT_, resulted in increased levels of PP2Acα-YC protein without affecting the levels of YN-Aα or endogenous Aα and PP2Acα (Fig. 6B). Consistent with the BiFC analysis, more complexes formed between PP2Acα-YC and YN-Aα or endogenous Aα in the presence of α4_WT_ compared to that in the presence of vector or α4_MUT_ (Fig. 6B). These findings demonstrate that α4 may not act as a competitor of the A subunit for binding PP2Ac in cells, but instead likely plays a protective role in stabilizing newly synthesized free C subunits prior to formation of a stable AC core enzyme complex [17], [21].

### Visualization of the trimeric PP2A holoenzyme complexes

**Figure 7:**
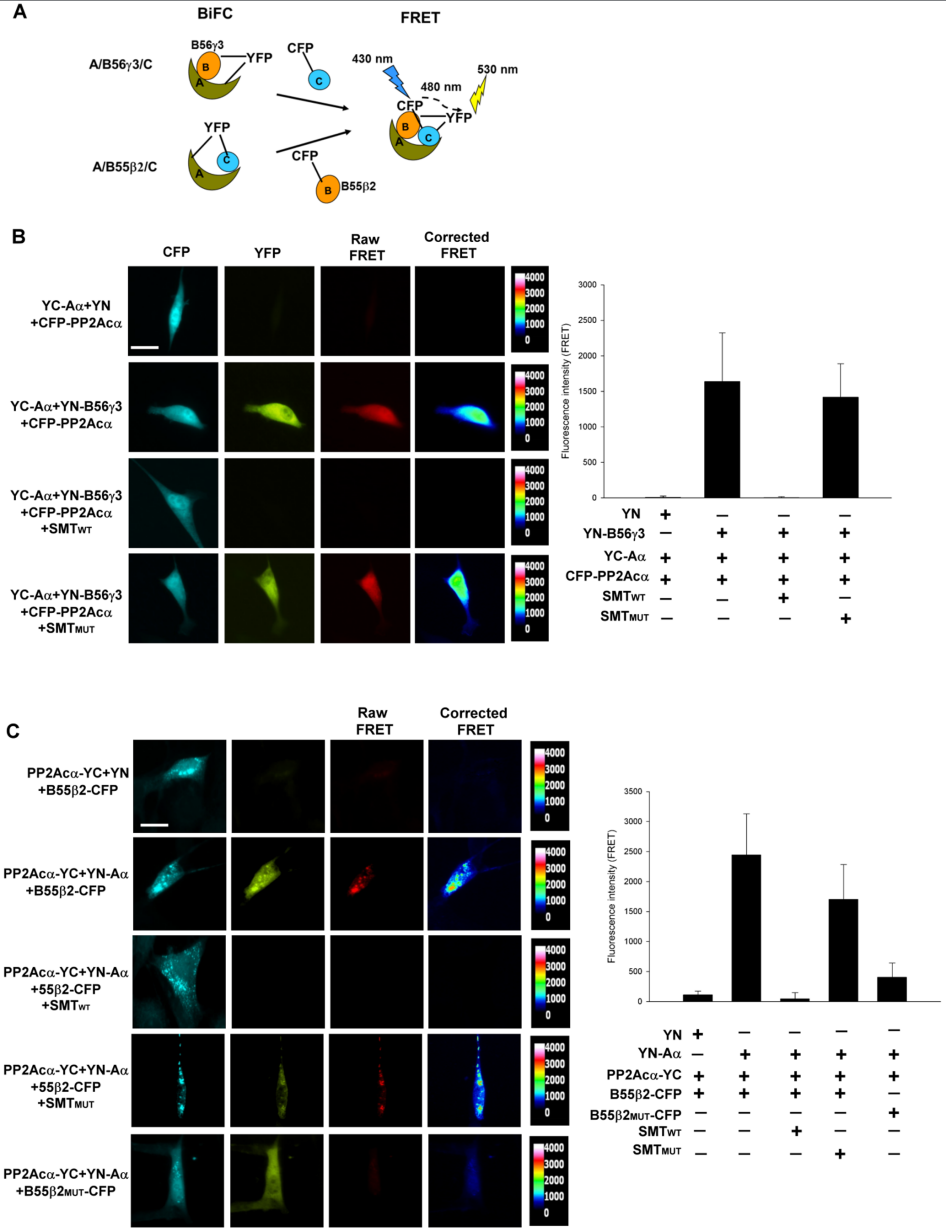
Visualization of the PP2A trimeric holoenzyme complexes Aα/B56γ3/Cα and Aα/B55β2/Cα in cells by BiFC-FRET. (**A**) The design of BiFC-FRET analysis of Aα/B56γ3/Cα and Aα/B55β2/Cα is shown. YFP, which serves as a FRET acceptor, is reconstituted via BiFC of YC-Aα/YN-B56γ3 or PP2Acα-YC/YN-Aα, in ternary complex Aα/B56γ3/Cα or Aα/B55β2/Cα, respectively. FRET occurs when CFP-PP2Acα or B55β2-CFP, which serves as a FRET donor, associates with YC-Aα/YN-B56γ3 or YN-Aα/PP2Acα-YC BiFC complex, respectively. (**B**) NIH3T3 cells were transiently transfected with constructs encoding YC-Aα, CFP-PP2Acα and YN-vector or YN-B56γ3, in the presence of pCMV5-SMT_WT_, pCMV5-SMT_MUT_, or empty vector. Images of expression of CFP-PP2Acα, YFP (due to BiFC of YC-Aα and YN-B56γ3), and FRET between CFP and YFP were acquired by indicated filters. Corrected FRET images were generated and FRET intensity was assessed by AxioVision (Zeiss), as described in the “Methods and Materials”. Representative images are shown. Scale bars, 20 µm. Quantified data from one of two independent experiments with similar results are shown. Mean (±s.d.) of corrected FRET maximum intensity values of individual cells from at least 15 cells were measured for each group. (**C**) NIH3T3 cells were transiently transfected with constructs encoding PP2Acα-YC and YN-vector or YN-Aα, and B55β2-CFP in the presence of pCMV5-SMT_WT_, pCMV5-SMT_MUT_, or empty vector, or transfected with equal amounts of constructs harboring PP2Acα-YC, YN-Aα, and B55β2_MUT_-CFP. Representative images of expression of B55β2-CFP and B55β2_MUT_-CFP, YFP (due to BiFC of association of PP2Acα-YC and YN-Aα), and FRET between CFP and YFP were acquired by indicated filters as described above. Scale bars: 20 µm. Corrected FRET images were generated and FRET intensity was assessed as described earlier. Quantified data from one of two independent experiments with similar results are shown. At least 10 cells were measured for each group.

The PP2A heterotrimeric holoenzymes are the predominant forms of PP2A in cells, and have been purified from a number of different tissues and cell lines [4], [37], [38]. However, direct visualization of the holoenzyme complexes in cells has hitherto not been reported. After successfully utilizing BiFC to observe the association of two individual PP2A subunits in cells (Fig. 2), we next employed BiFC-FRET to visualize the trimeric PP2A holoenzyme complexes in cells (Fig. 7A). We first investigated the trimeric Aα/B56γ3/Cα complex by co-expressing YC-Aα and YN-B56γ3, which serves as a FRET acceptor when YFP is reconstituted via BiFC of YC-Aα/YN-B56γ3, together with CFP-PP2Acα, which serves as a FRET donor (Fig. 7A). As shown in Figure 7B, FRET occurred following co-expression of YC-Aα, YN-B56γ3, and CFP-PP2Acα, but no FRET was observed in cells expressing YC-Aα, YN-empty vector, and CFP-PP2Acα. These findings indicate that the YC-Aα/YN-B56γ3/CFP-PP2Acα heterotrimer can be formed in cells. The FRET mainly displayed a homogenous pattern throughout the entire cell, which is similar to the distribution patterns of B56γ3 (Fig. 1B) and BiFC complexes of Aα/B56γ3 and B56γ3/PP2Acα (Fig. 2C). The specificity of FRET between YC-Aα/YN-B56γ3 and CFP-PP2Acα was further verified by co-expression of SV40 SMT_WT_ or SMT_MUT_. Co-expression of SMT_WT_ abolished both BiFC (YFP) of the YC-Aα/YN-B56γ3 complex and FRET between the BiFC complex of YC-Aα/YN-B56γ3 and CFP-PP2Acα, but both BiFC (YFP) and FRET were retained when SMT_MUT_ was co-expressed (Fig. 7B).

To further establish the feasibility of using BiFC-FRET to visualize heterotrimeric PP2A complexes, we applied this method to visualize the Aα/B55β2/Cα complex. We co-expressed PP2Acα-YC and YN-Aα, which serves as a FRET acceptor when YFP is reconstituted via BiFC of PP2Acα-YC/YN-Aα, together with B55β2-CFP, which serves as a FRET donor (Fig. 7A). As shown in Figure 7C, FRET occurred when PP2Acα-YC, YN-Aα, and B55β2-CFP were co-expressed, and the pattern of FRET was largely punctate, similar to the distribution of B55β2-CFP (Fig. 7C), which is known to be a mitochondrially targeting subunit of PP2A [9]. The FRET was specific because co-expression of SMT_WT_, but not SMT_MUT_, along with PP2Acα-YC, YN-Aα, and B55β2-CFP resulted in no FRET (Figure 7C). In addition, we verified distribution pattern of Aα/B55β2/Cα by applying a mutant B55β2_MUT_ (RR168EE) [9], which is defective in binding with the A subunit. When B55β2_MUT_-CFP was co-expressed with PP2Acα-YC and YN-Aα, FRET did not occur (Fig. 7C), suggesting that the observed FRET was specifically due to formation of a ternary YN-Aα/B55β2-CFP/PP2Acα-YC complex. Together, these results demonstrate that the PP2A holoenzyme complexes, such as Aα/B56γ3/Cα and Aα/B55β2/Cα, can be visualized in cells by BiFC-FRET.

## Discussion

In this report, we utilized BiFC analysis to investigate dimeric interactions between canonical PP2A subunits (A, B, and C), and successfully visualized most of the possible dimeric complexes formed by these subunits in cells. We also employed BiFC to visualize non-canonical PP2Ac-α4 interactions. Furthermore, we employed BiFC-FRET to visualize two different heterotrimeric PP2A holoenzymes in cells. Our findings demonstrate that these fluorescence-based methods can be utilized to study interactions between subunits of both canonical and non-canonical PP2A complexes in cells.

The strengths of BiFC include high sensitivity, high signal-to-background ratio, and high spatial resolution of protein-protein interactions in intact cells [23]. Our BiFC analyses revealed differences in the subcellular localization of various B isoform-containing A/B complexes, which were markedly similar to the subcellular distribution patterns of individual B subunits (Figs. 1 and 2). Not surprisingly, BiFC analysis of B55βαβ (Fig. 1B), a domain swap mutant of B55β1 harboring 15 amino acids of B55α, also revealed a role for the highly divergent N-termini of B55α and B55β1 in determining the subcellular localization of these two highly related isoforms. In addition, BiFC analysis revealed that B56γ3, but not B55α, directed the A subunit to accumulate in the nucleus at the early S phase, which is consistent with our prior immunofluorescence and biochemical fractionation studies of individual subunits [29]. Our current findings not only demonstrate the high efficacy of using BiFC to assess the spatial distribution of dimeric interactions between PP2A subunits, but also provide the first direct imaging evidence indicating that the B subunit controls the subcellular localization of the PP2A holoenzyme.

The crystal structure of the trimeric PP2A Aα/B56γ/PP2Acα complex demonstrated extensive interactions between B56γ and PP2Acα subunits [39], [40], but our BiFC analysis of PP2Acα association with B55β1, B55δ, or B56γ3 showed that the interaction between the B and PP2Acα subunits is relatively inefficient without co-expression of the A subunit (S6-S8 Figs. and Fig. 2D). Co-expression of Aα markedly facilitated BiFC complexes formed between B55β1or B56γ3 and PP2Acα subunits (S6-S8 Figs. and Fig. 2D). The inefficient association between PP2Acα-YC and YN-B subunits in the absence of ectopically-expressed Aα may be partly attributed to limited availability of endogenous A subunit, as the levels of endogenous PP2A A and C subunits are known to be tightly controlled in mammalian cells [41]–[43]. The ectopic Aα likely promotes the assembly of the ternary complex when endogenous A subunits are limiting.

The presence of the fluorescent protein fragment at the C-terminus of PP2Acα-YC may prevent it from undergoing carboxymethylation, which is required for the recruitment of some B subunits [22], especially B55 family members, into the holoenzyme complex. Given that the non-carboxymethylated PP2Acα exhibits lower affinity for select B subunits [44], it is possible that the near irreversible association of two halves of YFP [23] helps tether the PP2Acα-YC and YN-B subunits in the presence of ectopic Aα subunit. In addition, it is possible that the binding to YN-Aα stabilizes the conformation of non-carboxymethylated PP2Acα-YC to increase the binding affinity for B55 subunits. Although B55δ shares 83% identity with B55β1, in contrast to B55β1, no PP2Acα/B55δ BiFC complexes were found regardless of whether or not Aα was co-expressed. The inability to detect PP2Acα/B55δ BiFC complexes could be the result of an unfavorable conformation for reconstituting fluorophore when PP2Acα associates with B55δ, since no obvious differences in expression levels of YN-B55β1 and YN-B55δ were detected, and YN-B55δ was found to bind as efficiently as YN-B55β1 to HA-PP2Acα-YC (S9 Fig.). Our collective studies clearly demonstrate the scaffolding role of the A subunit in recruiting B and C subunits to form a mature PP2A holoenzyme.

In addition to the canonical PP2A trimeric holoenzyme complex, we were able to visualize non-canonical PP2Acα/α4 complexes in cells by BiFC (Fig. 5). Our results demonstrate increased formation of dimeric AC complexes in the presence of α4, which can be attributed to elevated PP2Ac levels following α4 overexpression (Fig. 6). These findings are in agreement with the PP2Ac stabilizing role of α4 [17], [19]–[21].

BiFC and FRET have both been used to detect protein-protein interactions and visualize the localization of protein complexes in cells [23], [25]. Since the formation of PP2A complexes is postulated to be highly dynamic [22], we coupled BiFC with FRET, which measures instantaneous association and dissociation of multi-molecules [25], [45]. The combination of BiFC and FRET (BiFC-FRET) [27] allowed us to visualize two PP2A complexes in cells. The FRET signal for YC-Aα/YN-B56γ3/CFP-PP2Acα complexes closely mirrored the immunostaining pattern of B56γ3 (compare Fig. 7B and Fig. 1C), with the highest FRET found surrounding and in the nucleus, which is consistent with prior reports showing that a subset of B56γ3 is localized to the Golgi apparatus or enriched in the nucleus [11], [29], [46] (Fig. 7B). In contrast, the FRET signal for YN-Aα/B55β2-CFP/PP2Acα-YC complexes displayed a punctate, mitochondria-like distribution pattern. SV40 SMT is thought to displace the B subunit from the holoenzyme by binding to common HEAT repeats of the A subunit [34], [47]. Interestingly, we found that SV40 SMT also disrupts the BiFC between YN-Aα and PP2Acα-YC (Fig. 7C). Since SV40 SMT forms stable complexes with the AC core enzyme [47], we propose that the SMT-induced conformational change of the AC core enzyme is different from that made following B55β2 binding, which does not disrupt the BiFC between YN-Aα and PP2Acα-YC (Fig. 7C).

PP2A regulates myriad cellular functions, which is owing to the structural complexity of the PP2A holoenzymes. The holoenzyme complexes are thought to act on their substrates in a spatial and temporal manner, and are proposed to dynamically assemble and disassemble its components in response to environmental cues. Efforts to confirm prior models of PP2A regulatory processes have been hampered by the lack of tools to visualize distinct PP2A holoenzymes in cells. In this report, we demonstrate the successful application of BiFC-FRET to visualize two PP2A holoenzyme complexes in cells. We anticipate that this approach can be promptly applied to monitor changes in the localization of specific PP2A holoenzymes in real time. Moreover, by assessing the FRET of various ternary complexes (e.g., YC-PP2Ac/YN-A/B-CFP), we believe that this approach will allow us to study the dynamics of B subunit assembly with the AC core enzyme.

## Conclusions

BiFC was used to provide the first direct cell imaging evidence that the regulatory B subunit dictates the subcellular localization of the PP2A heterotrimeric holoenzyme, and that the A subunit functions as a scaffolding protein for assembly of a holoenzyme. In addition, our BiFC analyses indicate that α4 may not act as a competitor of the A subunit for binding C subunits in cells, but instead stabilizes newly synthesized free C subunits for subsequent integration into active AC or ABC complexes. The BiFC-FRET system is a promising approach for visualizing real-time dynamics of the subcellular distribution of individual PP2A holoenzymes in live cells.

## Supporting Information

S1 Fig **BiFC analysis of various combinations of paired BiFC expression constructs encoding YN- or YC-fused Aα and YC- or YN-fused PP2Acα.** Equal amounts of BiFC expression constructs encoding YN- or YC-fused Aα and YC- or YN-fused PP2Acα were co-transfected into NIH3T3 cells, and 24 h after transfection, YFP signals due to BiFC of paired YN- or YC-fused Aα and PP2Acα were measured by fluorescence microscopy. DAPI was applied for staining of nuclei. Scale bar: 50 µm.
(PDF)

S2 Fig **BiFC analysis of various combinations of paired BiFC expression constructs encoding YN- or YC-fused Aα and YC- or YN-fused B55α.** Equal amounts of BiFC expression constructs encoding YN- or YC-fused Aα and YC- or YN-fused B55α were co-transfected into NIH3T3 cells, and 24 h after transfection, YFP signals due to BiFC of paired YN- or YC-fused Aα and B55α were measured by fluorescence microscopy. DAPI was applied for staining of nuclei. Scale bar: 50 µm.
(PDF)

S3 Fig **BiFC analysis of various combinations of paired BiFC expression constructs encoding YN- or YC-fused Aα and YC- or YN-fused B55β1.** Equal amounts of BiFC expression constructs encoding YN- or YC-fused Aα and YC- or YN-fused B55β1 were co-transfected into NIH3T3 cells, and 24 h after transfection, YFP signals due to BiFC of paired YN- or YC-fused Aα and B55β1were measured by fluorescence microscopy. DAPI was applied for staining of nuclei. Scale bar: 50 µm.
(PDF)

S4 Fig **BiFC analysis of various combinations of paired BiFC expression constructs encoding YN- or YC-fused Aα and YC- or YN-fused B55βαβ.** Equal amounts of BiFC expression constructs encoding YN- or YC-fused Aα and YC- or YN-fused B55βαβ were co-transfected into NIH3T3 cells, and 24 h after transfection, YFP signals due to BiFC of paired YN- or YC-fused Aα and B55βαβ were measured by fluorescence microscopy. DAPI was applied for staining of nuclei. Scale bar: 50 µm.
(PDF)

S5 Fig **Co-immunoprecipitation of BiFC complexes of PP2A/Aα and various B subunits.** (**A**) Lysates of NIH3T3 cells co-transfected with pCMV-HA-PP2A/Aα-YFPC and pcDNAI-YFPN-B55αHA or empty vector were immunoprecipitated (IP) by a pan-anti-B55 antibody or by preimmune IgG as a control, and the immunocomplexes were analyzed by SDS-PAGE and Western blotting by specific anti-PP2A/Aα, anti-HA, and anti-PP2Acα antibodies. The asterisk indicates YFPN-B55αHA. (**B–E**) Lysates of NIH3T3 cells co-transfected with pCMV-HA-PP2A/Aα-YFPC and pcDNAI-YFPN-FLAG-B55β1, pcDNAI-YFPN- FLAG-B55β2, pcDNAI-YFPN-FLAG-B55δ, pcDNAI-YFPN-FLAG-B55βαβ, or empty vector were immunoprecipitated (IP) by anti-FLAG-Sepharose, and the immunocomplexes were analyzed by SDS-PAGE and Western blotting (WB) by specific anti-PP2A/Aα, anti-FLAG, and anti-PP2Acα antibodies. (**F**) Lysates of NIH3T3 cells co-transfected with pcDNAI-YFPC-PP2A/Aα and pcDNAI-YFPN-B56γ3HA or empty vector were immunoprecipitated (IP) by anti-HA antibody, and the immunocomplexes were analyzed by SDS-PAGE and Western blotting by specific anti-PP2A/Aα, anti-HA, and anti-PP2Acα antibodies.
(PDF)

S6 Fig **BiFC analysis of various combinations of paired BiFC expression constructs encoding YN- or YC-fused PP2Acα and YC- or YN-fused B55β1with or without co-expression of 6myc-PP2A/Aα.** Equal amounts of BiFC expression constructs encoding YN- or YC-fused PP2Acα and YC- or YN-fused B55β1 with or without equal amounts of pCA2-6myc-PP2A/Aα or vector were co-transfected into NIH3T3 cells, and 24 h after transfection, YFP signals due to BiFC were measured by direct fluorescence microscopy and expression of 6myc-PP2A/Aα was confirmed by indirect immunofluorescence using anti-Myc tag antibody in conjunction with Cy3-conjugated secondary antibody. DAPI was applied for staining of nuclei. Scale bar: 50 µm.
(PDF)

S7 Fig **BiFC analysis of various combinations of paired BiFC expression constructs encoding YN- or YC-fused PP2Acα and YC- or YN-fused B56γ3 with or without co-expression of 6myc-PP2A/Aα.** Equal amounts of BiFC expression constructs encoding YN- or YC-fused PP2Acα and YC- or YN-fused B56γ3 with or without equal amounts of pCA2-6myc-PP2A/Aα or vector were co-transfected into NIH3T3 cells, and 24 h after transfection, YFP signals due to BiFC were measured by direct fluorescence microscopy and expression of 6myc-PP2A/Aα was confirmed by indirect immunofluorescence using anti-Myc tag antibody in conjunction with Cy3-conjugated secondary antibody. DAPI was applied for staining of nuclei. Scale bar: 50 µm.
(PDF)

S8 Fig **BiFC analysis of various combinations of paired BiFC expression constructs encoding YN- or YC-fused PP2Acα and YC- or YN-fused B55δ with or without co-expression of 6myc-PP2A/Aα.** Equal amounts of BiFC expression constructs encoding YN- or YC-fused PP2Acα and YC- or YN-fused B55δ with or without equal amounts of pCA2-6myc-PP2A/Aα or vector were co-transfected into NIH3T3 cells, and 24 h after transfection, YFP signals due to BiFC were measured by direct fluorescence microscopy and expression of 6myc-PP2A/Aα was confirmed by indirect immunofluorescence using anti-Myc tag antibody in conjunction with Cy3-conjugated secondary antibody. DAPI was applied for staining of nuclei. Scale bar: 50 µm.
(PDF)

S9 Fig **Co-immunoprecipitation of BiFC complexes of PP2Acα-YC and YN-B55β1 or YN-B55δ in the presence of 6myc-PP2A/Aα.** Lysates of NIH3T3 cells co-transfected with equal amounts of BiFC expression constructs encoding PP2Acα-YC and YN-B55β1 or YN- B55δ with or without pCA2-6myc-PP2A/Aα were immunoprecipitated by anti-HA antibody and the immunocomplexes were analyzed by SDS-PAGE and Western blotting by specific anti-GFP, anti-HA, and anti-Myc tag antibodies.
(PDF)

S10 Fig **BiFC between Aα-YC and YN-B55α, YN-fused B55β1, B55β2, B55βαβ, or B55δ and BiFC between YC-Aα and YN-B56γ3 is disrupted by SV40 SMT.** BiFC expression constructs encoding Aα-YC and YN-fused B55α, B55β1, B55β2, B55βαβ, or B55δ and BiFC expression constructs encoding YC-Aα and YN-B56γ3 with pCMV5 SMT_WT_, -SMT_MUT_, or empty vector with a 1∶1∶2 ratio (Aα-YC or YC-Aα:YN-B:pCMV5-SMT_WT_, pCMV5-SMT_MUT_, or empty vector  = 1∶1∶2), were transfected into NIH3T3 cells, and 24 h after transfection, YFP signals due to BiFC were measured by fluorescence microscopy. Expression of HA-tagged YN-fused B55α, B55β1, B55β2, B55βαβ, B55δ, or B56γ3 was confirmed using anti-HA antibody by indirect immunofluorescence microscopy as described earlier. DAPI was applied for staining of nuclei. Scale bar: 50 µm.
(PDF)

S1 Table **Plasmids used in this study.**
(PDF)

S2 Table **Primers used in this study.**
(PDF)

S1 Text **Construction of expression plasmids.**
(PDF)
