# Supplementary material for: Visualization of Subunit Interactions and Ternary Complexes of Protein Phosphatase 2A in Mammalian Cells
Source: PLoS One. 2014 Dec 23;9(12):e116074. doi: 10.1371/journal.pone.0116074 (PMC4275284; doi:10.1371/journal.pone.0116074)
Supplement: S2 Fig — BiFC analysis of various combinations of paired BiFC expression constructs encoding YN- or YC-fused Aα and YC- or YN-fused B55α. Equal amounts of BiFC expression constructs encoding YN- or YC-fused Aα and YC- or YN-fused B55α were co-transfected into NIH3T3 cells, and 24 h after transfection, YFP signals due to BiFC of paired YN- or YC-fused Aα and B55α were measured by fluorescence microscopy. DAPI was applied for staining of nuclei. Scale bar: 50 µm. (PDF) [file pone.0116074.s002.pdf]

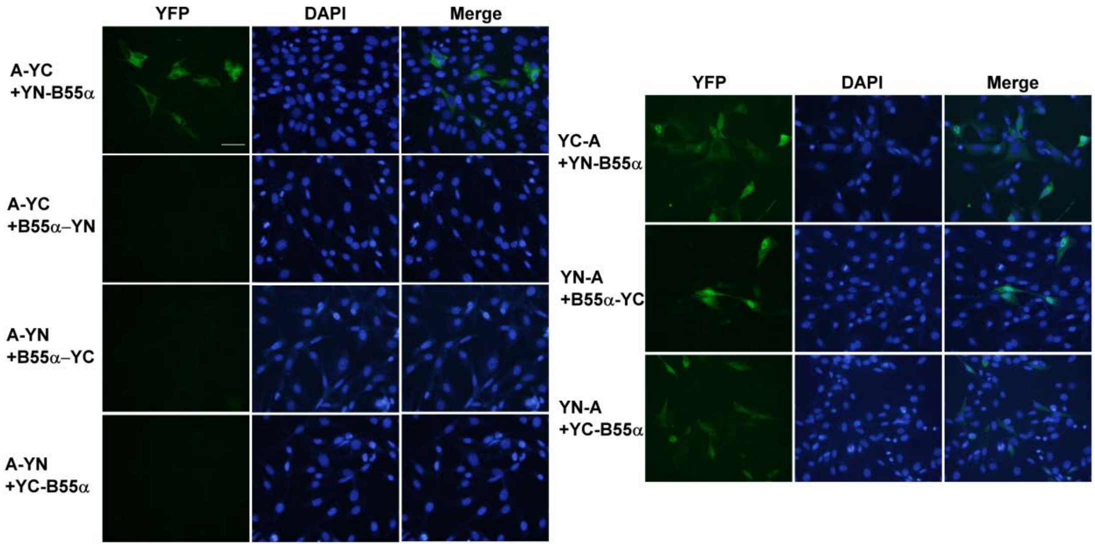

Figure S2. BiFC analysis of various combinations of paired BiFC expression constructs encoding YN- or YC-fused Aα and YC- or YN-fused B55α. Equal amounts of BiFC expression constructs encoding YN- or YC-fused Aα and YC- or YN-fused B55α were co-transfected into NIH3T3 cells, and 24 h after transfection, YFP signals due to BiFC of paired YN- or YC-fused Aα and B55α were measured by fluorescence microscopy. DAPI was applied for staining of nuclei. Scale bar: 50 μm.
