# Supplementary material for: Visualization of Subunit Interactions and Ternary Complexes of Protein Phosphatase 2A in Mammalian Cells
Source: PLoS One. 2014 Dec 23;9(12):e116074. doi: 10.1371/journal.pone.0116074 (PMC4275284; doi:10.1371/journal.pone.0116074)
Supplement: S5 Fig — Co-immunoprecipitation of BiFC complexes of PP2A/Aα and various B subunits. (A) Lysates of NIH3T3 cells co-transfected with pCMV-HA-PP2A/Aα-YFPC and pcDNAI-YFPN-B55αHA or empty vector were immunoprecipitated (IP) by a pan-anti-B55 antibody or by preimmune IgG as a control, and the immunocomplexes were analyzed by SDS-PAGE and Western blotting by specific anti-PP2A/Aα, anti-HA, and anti-PP2Acα antibodies. The asterisk indicates YFPN-B55αHA. (B–E) Lysates of NIH3T3 cells co-transfected with pCMV-HA-PP2A/Aα-YFPC and pcDNAI-YFPN-FLAG-B55β1, pcDNAI-YFPN- FLAG-B55β2, pcDNAI-YFPN-FLAG-B55δ, pcDNAI-YFPN-FLAG-B55βαβ, or empty vector were immunoprecipitated (IP) by anti-FLAG-Sepharose, and the immunocomplexes were analyzed by SDS-PAGE and Western blotting (WB) by specific anti-PP2A/Aα, anti-FLAG, and anti-PP2Acα antibodies. (F) Lysates of NIH3T3 cells co-transfected with pcDNAI-YFPC-PP2A/Aα and pcDNAI-YFPN-B56γ3HA or empty vector were immunoprecipitated (IP) by anti-HA antibody, and the immunocomplexes were analyzed by SDS-PAGE and Western blotting by specific anti-PP2A/Aα, anti-HA, and anti-PP2Acα antibodies. (PDF) [file pone.0116074.s005.pdf]

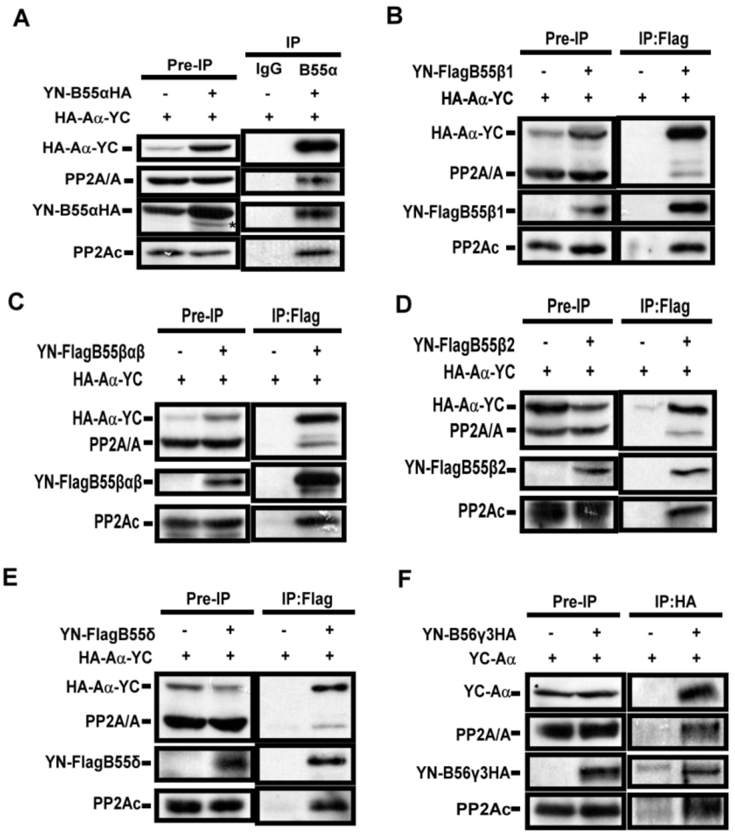

Figure S5. Co-immunoprecipitation of BiFC complexes of PP2A/Aα and various B subunits. (A) Lysates of NIH3T3 cells co-transfected with pCMV-HA-PP2A/Aα-YFPC and pcDNAI-YFPN-B55αHA or empty vector were immunoprecipitated (IP) by a pan-anti-B55 antibody or by preimmune IgG as a control, and the immunocomplexes were analyzed by SDS-PAGE and Western blotting by specific anti-PP2A/Aα, anti-HA, and anti-PP2Acα antibodies. The asterisk indicates YFPN-B55αHA. (B-E) Lysates of NIH3T3 cells co-transfected with pCMV-HA-PP2A/Aα-YFPC and pcDNAI-YFPN-FLAG-B55β1, pcDNAI-YFPN-FLAG-B55β2, pcDNAI-YFPN-FLAG-B55δ, pcDNAI-YFPN-FLAG-B55βαβ, or empty vector were immunoprecipitated (IP) by anti-FLAG-Sepharose, and the immunocomplexes were analyzed by SDS-PAGE and Western blotting (WB) by specific anti-PP2A/Aα, anti-FLAG, and anti-PP2Acα antibodies. (F) Lysates of NIH3T3 cells co-transfected with pcDNAI-YFPC-PP2A/Aα and pcDNAI-YFPN-B56γ3HA or empty vector were immunoprecipitated (IP) by anti-HA antibody, and the immunocomplexes were analyzed by SDS-PAGE and Western blotting by specific anti-PP2A/Aα, anti-HA, and anti-PP2Acα antibodies.
