# Supplementary material for: Visualization of Subunit Interactions and Ternary Complexes of Protein Phosphatase 2A in Mammalian Cells
Source: PLoS One. 2014 Dec 23;9(12):e116074. doi: 10.1371/journal.pone.0116074 (PMC4275284; doi:10.1371/journal.pone.0116074)
Supplement: S8 Fig — BiFC analysis of various combinations of paired BiFC expression constructs encoding YN- or YC-fused PP2Acα and YC- or YN-fused B55δ with or without co-expression of 6myc-PP2A/Aα. Equal amounts of BiFC expression constructs encoding YN- or YC-fused PP2Acα and YC- or YN-fused B55δ with or without equal amounts of pCA2-6myc-PP2A/Aα or vector were co-transfected into NIH3T3 cells, and 24 h after transfection, YFP signals due to BiFC were measured by direct fluorescence microscopy and expression of 6myc-PP2A/Aα was confirmed by indirect immunofluorescence using anti-Myc tag antibody in conjunction with Cy3-conjugated secondary antibody. DAPI was applied for staining of nuclei. Scale bar: 50 µm. (PDF) [file pone.0116074.s008.pdf]

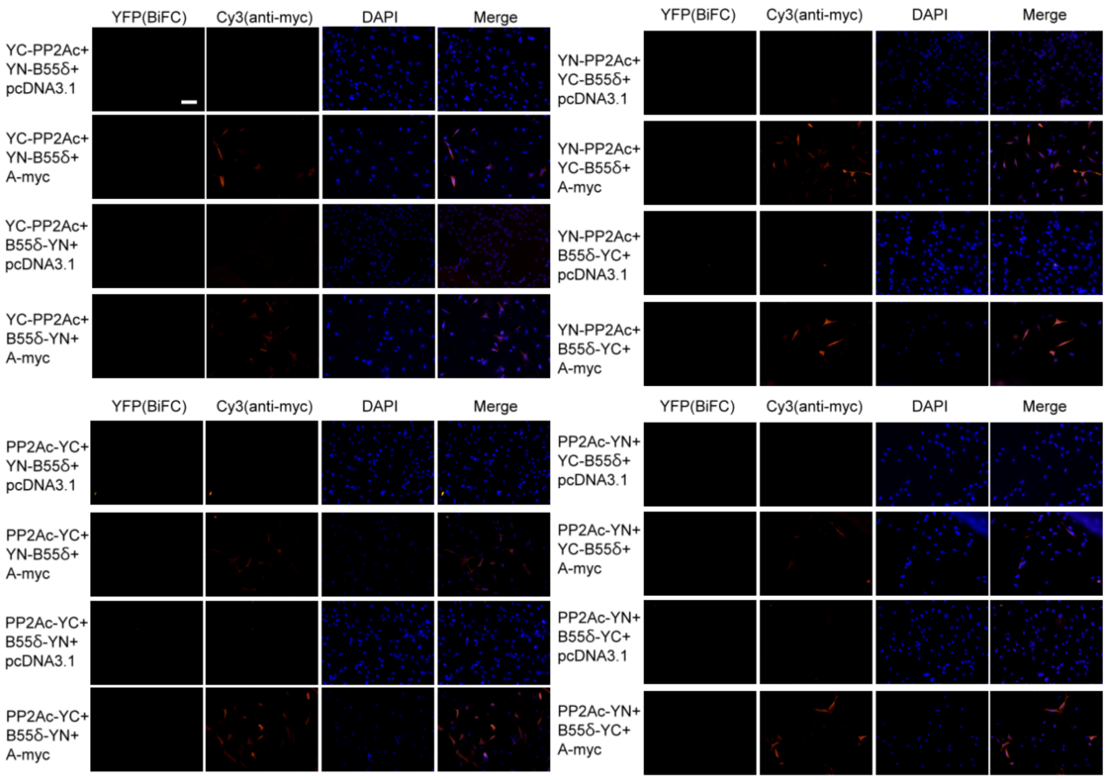

Figure S8. BiFC analysis of various combinations of paired BiFC expression constructs encoding YN- or YC-fused PP2Acα and YC- or YN-fused B55δ with or without co-expression of 6myc-PP2A/Aα. Equal amounts of BiFC expression constructs encoding YN- or YC-fused PP2Acα and YC- or YN-fused B55δ with or without equal amounts of pCA2-6myc-PP2A/Aα or vector were co-transfected into NIH3T3 cells, and 24 h after transfection, YFP signals due to BiFC were measured by direct fluorescence microscopy and expression of 6myc-PP2A/Aα was confirmed by indirect immunofluorescence using anti-Myc tag antibody in conjunction with Cy3-conjugated secondary antibody. DAPI was applied for staining of nuclei. Scale bar: 50 μm.
