# Supplementary material for: Visualization of Subunit Interactions and Ternary Complexes of Protein Phosphatase 2A in Mammalian Cells
Source: PLoS One. 2014 Dec 23;9(12):e116074. doi: 10.1371/journal.pone.0116074 (PMC4275284; doi:10.1371/journal.pone.0116074)
Supplement: S9 Fig — Co-immunoprecipitation of BiFC complexes of PP2Acα-YC and YN-B55β1 or YN-B55δ in the presence of 6myc-PP2A/Aα. Lysates of NIH3T3 cells co-transfected with equal amounts of BiFC expression constructs encoding PP2Acα-YC and YN-B55β1 or YN- B55δ with or without pCA2-6myc-PP2A/Aα were immunoprecipitated by anti-HA antibody and the immunocomplexes were analyzed by SDS-PAGE and Western blotting by specific anti-GFP, anti-HA, and anti-Myc tag antibodies. (PDF) [file pone.0116074.s009.pdf]

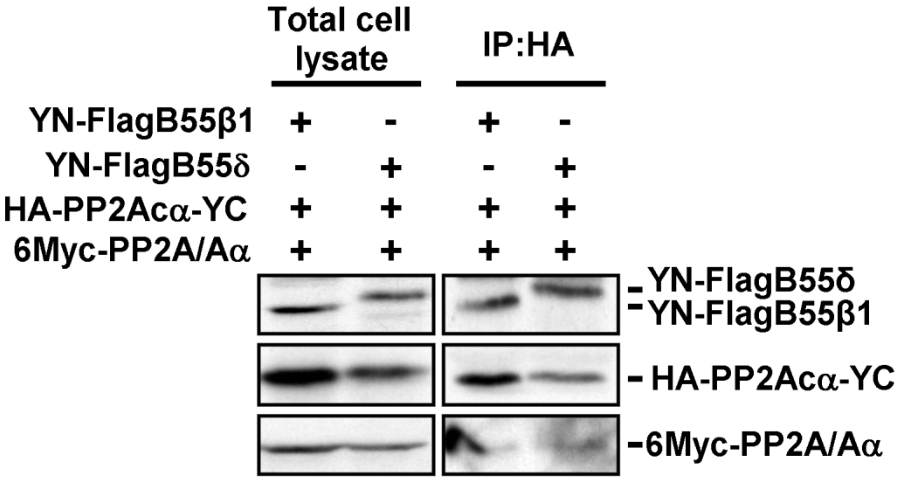


**Figure S9. Co-immunoprecipitation of BiFC complexes of PP2Acα-YC and YN-B55β1 or YN-B55δ in the presence of 6myc-PP2A/Aα.** Lysates of NIH3T3 cells co-transfected with equal amounts of BiFC expression constructs encoding PP2Acα-YC and YN-B55β1 or YN- B55δ with or without pCA2-6myc-PP2A/Aα were immunoprecipitated by anti-HA antibody and the immunocomplexes were analyzed by SDS-PAGE and Western blotting by specific anti-GFP, anti-HA, and anti-Myc tag antibodies.
