# Supplementary material for: Visualization of Subunit Interactions and Ternary Complexes of Protein Phosphatase 2A in Mammalian Cells
Source: PLoS One. 2014 Dec 23;9(12):e116074. doi: 10.1371/journal.pone.0116074 (PMC4275284; doi:10.1371/journal.pone.0116074)
Supplement: S10 Fig — BiFC between Aα-YC and YN-B55α, YN-fused B55β1, B55β2, B55βαβ, or B55δ and BiFC between YC-Aα and YN-B56γ3 is disrupted by SV40 SMT. BiFC expression constructs encoding Aα-YC and YN-fused B55α, B55β1, B55β2, B55βαβ, or B55δ and BiFC expression constructs encoding YC-Aα and YN-B56γ3 with pCMV5 SMTWT, -SMTMUT, or empty vector with a 1∶1∶2 ratio (Aα-YC or YC-Aα:YN-B:pCMV5-SMTWT, pCMV5-SMTMUT, or empty vector = 1∶1∶2), were transfected into NIH3T3 cells, and 24 h after transfection, YFP signals due to BiFC were measured by fluorescence microscopy. Expression of HA-tagged YN-fused B55α, B55β1, B55β2, B55βαβ, B55δ, or B56γ3 was confirmed using anti-HA antibody by indirect immunofluorescence microscopy as described earlier. DAPI was applied for staining of nuclei. Scale bar: 50 µm. (PDF) [file pone.0116074.s010.pdf]

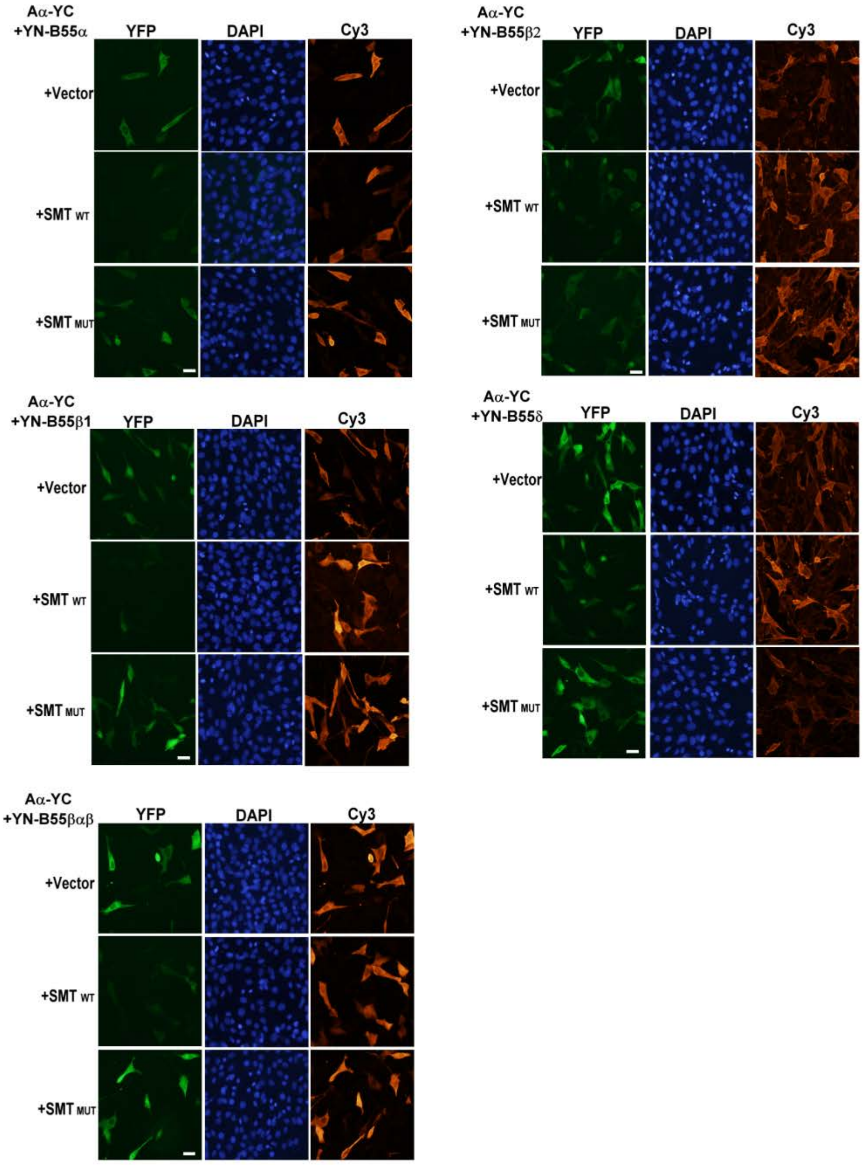

Figure S10. BiFC between Aα-YC and YN-B55α, YN-fused B55β1, B55β2, B55βαβ, or B55δ and BiFC between YC-Aα and YN-B56γ3 is disrupted by SV40 SMT. BiFC expression constructs encoding Aα-YC and YN-fused B55α, B55β1, B55β2, B55βαβ, or B55δ and BiFC expression constructs encoding YC-Aα and YN-B56γ3 with pCMV5 SMT_{WT}, -SMT_{MUT}, or empty vector with a 1:1:2 ratio (Aα-YC or YC-Aα:YN-B:pCMV5-SMT_{WT}, pCMV5-SMT_{MUT}, or empty vector=1:1:2), were transfected into NIH3T3 cells, and 24 h after transfection, YFP signals due to BiFC were measured by fluorescence microscopy. Expression of HA-tagged YN-fused B55α, B55β1, B55β2, B55βαβ, B55δ, or B56γ3 was confirmed using anti-HA antibody by indirect immunofluorescence microscopy as described earlier. DAPI was applied for staining of nuclei. Scale bar: 50 μm.
