# Supplementary material for: Visualization of Subunit Interactions and Ternary Complexes of Protein Phosphatase 2A in Mammalian Cells
Source: PLoS One. 2014 Dec 23;9(12):e116074. doi: 10.1371/journal.pone.0116074 (PMC4275284; doi:10.1371/journal.pone.0116074)
Supplement: S1 Table — Plasmids used in this study. (PDF) [file pone.0116074.s011.pdf]

Table S1. Plasmids used in this study.

Plasmid	Description	Reference
pcDNA3.1/Zeo(+)-B55α-HA	Cloned by PCR-based method; see the supplementary materials and methods	this study
pcDNA3.1/Zeo(+)-B55β-HA	Cloned by PCR-based method; see the supplementary materials and methods	this study
pcDNA3.1/Zeo(+)-B55βαβ-HA	Cloned by PCR-based method; see the supplementary materials and methods	this study
pcDNA3.1/Zeo(+)-B55β2-HA	Cloned by PCR-based method; see the supplementary materials and methods	this study
pcDNA5/TO-Flag-B55β2mut (RR168EE)	Provided by Dr. Stefan Strack, University of Iowa, USA	Dagda *et al.*, 2003
pcDNA3.1/Zeo(+)-B55δ-HA	Cloned by PCR-based method; see the supplementary materials and methods	this study
pcDNA3.1/Zeo(+)-B56γ3-HA	Cloned by PCR-based method	Lee *et al.*, 2010
pHM6-HA-PP2Acα	pHM6 with HA-tagged human PP2Acα	Chuang *et al.*, 2000
pcDNA5/TO-Flag-α4	pcDNA5/TO with FLAG-tagged α4	McConnell et al., 2010
pcDNA5/TO-Flag-α4mut(R155E/K158D)	pcDNA5/TO with FLAG-tagged α4(R155E/K158D)	McConnell et al., 2010
pCMV5-small T wt	pCMV5 with SV40 Small t antigen	Sontag et al., 1993
pCMV5-small T mut	pCMV5 with mutant SV40 Small t antigen (1-110)	Sontag et al., 1993
pcDNAI/Amp YFP-N (1-158)	pcDNAI/Amp with YFP (1-158)	Hynes et al., 2004
pcDNAI/Amp YFP-C (159-238)	pcDNAI/Amp with YFP (159-238)	Hynes et al., 2004
pBiFC-bFosYC155	pCMV-HA-YFP-C(155-238) with bFos	Hu et al., 2002
pBiFC-bJunYN155	pFLAG-CMV2-YFP-N (1-154) with bJun	Hu et al., 2002
pFLAG-CMV2-YN	Derived from pBiFC-bFosYC155 by cutting out bFos	this study
pCMV-HA-YC	Derived from pBiFC-bFosYC155 by cutting out bFos	this study
pcDNAI-YN Aα (encoding YN-Aα)	pcDNAI/Amp YN with PP2A/Aα; see the supplementary materials and methods	this study
pcDNAI-YC Aα(encoding YC-Aα)	pcDNAI/Amp YN with PP2A/Aα; see the supplementary materials and methods	this study
pcDNAI-YN B55α-HA (encoding YN-B55α)	pcDNAI/Amp YN with HA-tagged PP2A/B55α; see the supplementary materials and methods	this study

pcDNAI-YC B55α-HA(encoding YC-B55α)	pcDNAI/Amp YC with HA-tagged PP2A/B55α; see the supplementary materials and methods	this study
pcDNAI-YN FLAG-B55β1(encoding YN -B55β1)	pcDNAI/Amp YN with FLAG-tagged PP2A/B55β1; see the supplementary materials and methods	this study
pcDNAI-YC FLAG-B55β1(encoding YC -B55β1)	pcDNAI/Amp YC with FLAG-tagged PP2A/B55β1; see the supplementary materials and methods	this study
pcDNAI-YN FLAG-B55β2(encoding YN -B55β2)	pcDNAI/Amp YN with FLAG-tagged PP2A/B55β2; see the supplementary materials and methods	this study
pcDNAI-YC FLAG-B55β2(encoding YC -B55β2)	pcDNAI/Amp YC with FLAG-tagged PP2A/B55β2; see the supplementary materials and methods	this study
pcDNAI-YN FLAG-B55βαβ(encoding YN-B55βαβ)	pcDNAI/Amp YN with FLAG-tagged PP2A/B55βαβ; see the supplementary materials and methods	this study
pcDNAI-YC FLAG-B55βαβ(encoding YC-B55βαβ)	pcDNAI/Amp YC with FLAG-tagged PP2A/B55βαβ; see the supplementary materials and methods	this study
pcDNAI-YN FLAG-B55β2mut(encoding YN-B55β2mut)	pcDNAI/Amp YN with FLAG-tagged PP2A/B55β2mut (RR168EE); see the supplementary materials and methods	this study
pcDNAI-YC FLAG-B55β2mut (encoding YC-B55β2mut)	pcDNAI/Amp YC with FLAG-tagged PP2A/B55β2mut (RR168EE); see the supplementary materials and methods	this study
pcDNAI-YN FLAG-B55δ(encoding YN -B55δ)	pcDNAI/Amp YN with FLAG-tagged PP2A/B55δ; see the supplementary materials and methods	this study
pcDNAI-YC FLAG-B55δ(encoding YC -B55δ)	pcDNAI/Amp YC with FLAG-tagged PP2A/B55δ; see the supplementary materials and methods	this study
pcDNAI-YN B56γ3-HA(encoding YN -B56γ3)	pcDNAI/Amp YN with HA-tagged PP2A/ B56γ3; see the supplementary materials and methods	this study

pcDNAI-YC-B56γ3-HA(encoding YC -B56γ3)	pcDNAI/Amp YN with HA-tagged PP2A/ B56γ3; see the supplementary materials and methods	this study
pcDNAI-YN-PP2Acα(encoding YN-PP2Acα)	pcDNAI/Amp YN with PP2Acα; see the supplementary materials and methods	this study
pcDNAI-YC-PP2Acα(encoding YC-PP2Acα)	pcDNAI/Amp YC with PP2Acα; see the supplementary materials and methods	this study
pCMV2-FLAG-Aα-YN(encoding Aα-YN)	pCMV2-FLAG-YN with PP2A Aα	this study
pCMV-HA-Aα-YC(encoding Aα-YC)	pCMV-HA-YC with PP2A Aα	this study
pCMV2-FLAG-B55β1-YN(encoding B55β1-YN)	pCMV2-FLAG-YN with PP2A B55β1	this study
pCMV-B55β1-HA-YC(encoding B55β1-YC)	pCMV-HA-YC with PP2A B55β1	this study
pCMV2-FLAG-B55βαβ-YN(encoding B55βαβ-YN)	pCMV2-FLAG-YN with PP2A B55βαβ	this study
pCMV-B55βαβ-HA-YC(encoding B55βαβ-YC)	pCMV-HA-YC with PP2A B55βαβ	this study
pCMV2-FLAG-B55β2-YN(encoding B55β2-YN)	pCMV2-FLAG-YN with PP2A B55β2	this study
pCMV-B55β2-HA-YC(encoding B55β2-YC)	pCMV-HA-YC with PP2A B55β2	this study
pCMV2-FLAG-B55β2mut-YN(encoding B55β2mut -YN)	pCMV2-FLAG-YN with PP2A B55β2mut(RR168EE)	this study
pCMV-B55β2mut -HA-YC(encoding B55β2mut-YC)	pCMV-HA-YC with PP2A B55β2 mut (RR168EE)	this study
pCMV2-FLAG-B55δ-YN(encoding B55δ-YN)	pCMV2-FLAG-YN with PP2A B55δ	this study
pCMV-HA-B55δ-YC(encoding B55δ-YC)	pCMV-HA-YC with PP2A B55δ	this study
pCMV2-FLAG-PP2Acα-YN(encoding PP2Acα-YN)	pCMV2-FLAG-YN with PP2Acα	this study
pCMV-HA-PP2Acα-YC(encoding PP2Acα-YC)	pCMV-HA-YC with PP2Acα	this study
pcDNAI/Amp YN-MYC-α4(encoding YN-α4)	pcDNAI/Amp YN with MYC-tagged α4	this study
pcDNAI/Amp YC-α4mut(encoding YC-α4 mut)	pcDNAI/Amp YN with MYC-tagged α4mut (R155E/K158D)	this study

pCMV-MYC-α4-YC(encoding α4-YC)	pCMV-HA-YC with MYC tagged-α4	this study
pCMV-MYC-α4mut-YC(encoding α4mut-YC)	pCMV-HA-YC with MYC tagged-α4 mut(R155E/K158D)	this study
pECFP-C1-PP2Acα(encoding CFP-PP2Acα)	pECFP-C1 with PP2Acα	This study
pECFP-N1-FLAG-B55β2(encoding CFP-B55β2)	pECFP-N1 with FLAG-tagged B55β2	This study
pECFP-N1-FLAG-B55β2mut(encoding CFP-B55β2mut)	pECFP-N1 with FLAG-tagged B55β2 mut (RR168EE)	This study

Table S1 References

1.Dagda RK, Zaucha JA, Wadzinski BE, Strack S (2003) A developmentally regulated, neuron-specific splice variant of the variable subunit Bbeta targets protein phosphatase 2A to mitochondria and modulates apoptosis. J Biol Chem. **278**:24976-24985

2. Lee TY, Lai TY, Lin SC, Wu CW, Ni IF, Yang YS, Hung LY, Law BK, Chiang CW (2010) The B56gamma3 regulatory subunit of protein phosphatase 2A (PP2A) regulates S phase-specific nuclear accumulation of PP2A and the G1 to S transition. J Biol Chem. **285**:21567-21580

3. Chuang E, Fisher TS, Morgan RW, Robbins MD, Duerr JM, Vander Heiden MG, Gardner JP, Hambor JE, Neveu MJ, Thompson CB (2000) The CD28 and CTLA-4 receptors associate with the serine/threonine phosphatase PP2A. Immunity **13**:313-322

4. McConnell JL, Watkins GR, Soss SE, Franz HS, McCorvey LR, Spiller BW, Chazin WJ, Wadzinski BE (2010) Alpha4 is a ubiquitin-binding protein that regulates protein serine/threonine phosphatase 2A ubiquitination. Biochemistry **49**:1713-1718

5. Sontag E, Fedorov S, Kamibayashi C, Robbins D, Cobb M, Mumby M (1993) The interaction of SV40 small tumor antigen with protein phosphatase 2A stimulates the map kinase pathway and induces cell proliferation. Cell **75**:887-897

6 Hynes TR, Tang L, Mervine SM, Sabo JL, Yost EA, Devreotes PN, Berlot CH (2004) Visualization of G protein betagamma dimers using bimolecular fluorescence complementation demonstrates roles for both beta and gamma in subcellular targeting. J Biol Chem. 279:30279-30286

7. Hu CD, Chinenov Y, Kerppola TK (2002) Visualization of interactions among bZIP and Rel family proteins in living cells using bimolecular fluorescence complementation. Mol Cell. **9**:789-98
