# Supplementary material for: Visualization of Subunit Interactions and Ternary Complexes of Protein Phosphatase 2A in Mammalian Cells
Source: PLoS One. 2014 Dec 23;9(12):e116074. doi: 10.1371/journal.pone.0116074 (PMC4275284; doi:10.1371/journal.pone.0116074)
Supplement: S2 Table — Primers used in this study. (PDF) [file pone.0116074.s012.pdf]

Table S2. Primers used in this study.

Plasmids	Forward primer	Reverse primer
pcDNAI-YN-Flag-B55β1	5’-GGCTCGAGGTACCAAGCTTGAATTCGACTACAAAGACGATGACGATAAA-3’	5’-GGTCTAGAGGACTAGTATTCAGTATG-3’
pcDNAI-YC-Flag-B55β1	5’-GGCTCGAGGTACCAAGCTTGAATTCGACTACAAAGACGATGACGATAAA-3’	5’-GGTCTAGAGGACTAGTATTCAGTATG-3’
pcDNAI-YN-Flag-B55βαβ	5’-GGCTCGAGGTACCAAGCTTGAATTCATGGAGGAGGACATTCAATGG-3’	5’-GGTCTAGAGGACTAGTATTCAGTATG-3’
pcDNAI-YC-Flag-B55βαβ	5’-GGCTCGAGGTACCAAGCTTGAATTCATGGAGGAGGACATTCAATGG-3’	5’-GGTCTAGAGGACTAGTATTCAGTATG-3’
pcDNAI-YN-Flag-B55β2<br>pcDNAI-YN-Flag-B55β2mut	5'-GGCTCGAGGGCCCACCAAGCTTATGAAATGCTTCTCTCGTTAC-3'	5'-GGTCTAGACTGCAGAATTCCACCACA-3'
pcDNAI-YC-Flag-B55β2<br>pcDNAI-YC-Flag-B55β2mut	5'-GGCTCGAGGGCCCACCAAGCTTATGAAATGCTTCTCTCGTTAC-3'	5'-GGTCTAGACTGCAGAATTCCACCACA-3'
pcDNAI-YN-Myc-α4<br>pcDNAI-YN-Myc-α4mut	5'-GGAGATCTGCCACCATGGAGCAAAAGCTCATTTCTGAAGAGGACTTGATGGCTGCTGAGGACGAGTTAC-3'	5'-GGGTCGACTCAGCCCATGTTCTGTCGG-3'
pcDNAI-YN-Flag-B55β_2<br>pcDNAI-YN-Flag-B55$\beta_2$mut	5'-GGCTCGAGGGCCCACCAAGCTTATGAAATGCTTCTCTCGTTAC-3'	5'-GGTCTAGACTGCAGAATTCCACCACA-3'
pcDNAI-YN-Myc-α4<br>pcDNAI-YN-Myc-α4mut	5'-GGAGATCTGCCACCATGGAGCAAAAGCTCATTTCTGAAGAGGACTTGATGGCTGCTGAGGACGAGTTAC-3'	5'-GGGTCGACTCAGCCCATGTTCTGTCGG-3'
pFLAG-CMV2-Aα-YN	5'-GGGAATTCGGCACGAGCCAAGAT-3'	5'-GGGCTAGCGGCAAGAGAGAGAACAGTCAG-3'
pFLAG-CMV2-Cα-YN	5'-GGGAATTCAATGGACGAGAAGGTGTTCAC-3'	5'-GGGCTAGCCAGGAAGTAGTCTGGGGTACG-3'
pFLAG-CMV2-B55δ-YN	5'-GGGAATTCAATGGCAGGAGCCGG-3'	5'-GGGCTAGCATTAATTTTGTCCTGGAATATATA-3'

pFLAG-CMV2-B55β2-YN	5'-GGCTCGAGGGCCCACCAAGCTTATGAAATGCTTCTCTCGTTAC-3'	5'-GGTCTAGAATTAACCTTGTCCTGGAATATATA-3'
pCMV-HA-Aα-YC	5'-GGGAATTCGGGCACGAGCCAAGATGGCAG-3'	5'-GGGGTACCGGCAAGAGAGAGAACAGTCAG-3'
pCMV-HA-Cα-YC	5'-GGGAATTCGGATGGACGAGAAGGTGTTCACCA-3'	5'-GGGGTACCCAGGAAGTAGTCTGGGGTACG-3'
pCMV-HA-B55δ-YC	5'-GGGAATTCGGATGGCAGGAGCCG-3'	5'-GGGGTACCATTAATTTTGTCCTGGAATATATA-3'
pCMV-HA-B55β2-YC	5'-GGCTCGAGGGCCCACCAAGCTTATGAAATGCTTCTCTCGTTAC-3'	5'-GGGGTACCAGCGTAGTCTGGGACGTCGTATGGGTAATTAACCTTGTCCTGGAATAT-3'
pCMV-Myc-α4-YC<br>pCMV-Myc-α4mut-YC	5'-GGGGGCCCGCCACCATGGAGCAAAAGCTCATTTCTGAAGAGGACTTGATGGCTGCTGAGGACGAGTTAC-3'	5'-GGGGTACCGCCCATGTTCTGTCGGTTC-3'
pECFP-N1-Flag-B55β2<br>pECFP-N1-Flag-B55β2mut	5'-GGAAGCTTGCGCCACCATGAAATGCTTCTC-3'	5'-GGGTCGACTGTTTATCGTCATCGTCTTTGTAGTCATTAACCTTGTCCTGGAATATATATAAG-3'
pECFP-C1-PP2Acα	5'-GGGGATCCATGGACGAGAAGGTGTTC-3’	5'-GGGAATTCCTTACAGGAAGTAGTCTGGGG-3'
