# Supplementary material for: Visualization of Subunit Interactions and Ternary Complexes of Protein Phosphatase 2A in Mammalian Cells
Source: PLoS One. 2014 Dec 23;9(12):e116074. doi: 10.1371/journal.pone.0116074 (PMC4275284; doi:10.1371/journal.pone.0116074)
Supplement: S1 Text — Construction of expression plasmids. (PDF) [file pone.0116074.s013.pdf]

Text S1. Construction of expression plasmids.

The original constructs harboring cDNAs encoding subunits of PP2A include pcDNA3.1/Zeo(+)-Aα, pCA2-6XMYC-Aα, pcDNA3.1/Zeo(+)-B55α-HA, pcDNA5/TO-Flag-B55β, pcDNA5/TO-Flag-B55βαβ, pcDNA5/TO-Flag-B55β2, pcDNA5/TO-Flag-B55β2mut (RR168EE) (a kind gift from Dr. Stefan Strack, Department of Pharmacology, University of Iowa, USA), pcDNA5/TO-Flag-B55δ, pcDNA3.1/Zeo(+)-B56γ3-HA, pHM6-Cα, pcDNA5/TO-Flag-α4, and pcDNA5/TO-Flag-α4mut. pCMV5-small T wt and pCMV5-small T mut are kind gifts of Dr. Estelle Sontag, University of Newcastle, Australia. BiFC expression vectors pcDNAI/Amp YFP (1-158) and pcDNAI/Amp YFP (159-238) were kind gifts of Dr. Catherine Berlot, Weis Center for Research, Geisinger Clinic, USA. BiFC expression vectors pFLAG-CMV2-YFPN (1-154) and pCMV2-HA-YFPC (155-238) were obtained by excising cDNAs of bFos and bJun from pBiFC-bFosYC155 and pBiFC-bJunYN155 (kind gifts form Dr. Tom Kerppola, HHMI, University of Michigan, USA), respectively. For constructing BiFC expression vectors encoding YFPN- and YFPC-fused to the N-terminus of Aα, cDNA of Aα was excised from pcDNA3.1/Zeo(+)-Aα by BamHI and NotI digestion, and subcloned into BglII and NotI sites of pcDNAI/Amp YFP (1-158) and pcDNAI/Amp YFP (159-238). For constructing BiFC expression vectors encoding YFPN- and YFPC-fused to the C-terminus of Aα, cDNA of Aα was amplified by PCR, digested with EcoRI and NheI, or EcoRI and KpnI, and subcloned into EcoRI and NheI sites of pFLAG-CMV2-YFPN or EcoRI and KpnI sites of pCMV2-HA-YFPC, respectively. For constructing BiFC expression vectors encoding YFPN- and YFPC-fused to the N-terminus of B55α, cDNA of B55α was excised from pcDNA3.1/Zeo(+)-B55α-HA by BglII and XhoI digestion, and then subcloned into BglII and XhoI sites of pcDNAI-YFPN and pcDNAI-YFPC. For constructing BiFC expression vectors encoding YFPN- and YFPC-fused to the N-terminus of B55δ, cDNA of B55δ was excised from pcDNA5/TO-Flag-B55δ by BamHI and XhoI digestion, and then subcloned into BglII and XhoI sites of pcDNAI-YFPN and pcDNAI-YFPC. For constructing BiFC expression vectors encoding YFPN- and YFPC-fused to the N-terminus of B56γ3, cDNA of B56γ3 was excised from pcDNA3.1/Zeo(+)-B56γ3-HA by BamHI and XhoI, and then subcloned into BglII and XhoI sites of pcDNAI-YFPN and pcDNAI-YFPC. For constructing BiFC expression vectors encoding YFPN- and YFPC-fused to the N-terminus of B55β2, B55β2mut (RR168EE), and B55βαβ, cDNAs of B55β2, B55β2mut (RR168EE), and B55βαβ were amplified by PCR using plasmids described earlier and a XhoI site on the 5’ end and a XbaI site on the 3’ end were introduced, then subcloned into XhoI and XbaI sites of pcDNAI-YFPN and pcDNAI-YFPC. For constructing BiFC

expression vectors encoding YFPN- and YFPC-fused to the N-terminus of α4 or α4mut, cDNAs of α4 or α4mut were amplified by PCR using plasmids described earlier, and a myc-epitope tag and a BglII site on the 5' end, and a SalI site on the 3' end were introduced, and subcloned into BglII and XhoI sites of pcDNAI-YFPN and pcDNAI-YFPC. For constructing BiFC expression vectors encoding YFPN- and YFPC-fused to the C-terminus of Aα, Cα, and B55δ, cDNAs of Aα, Cα, and B55δ were amplified by PCR using plasmids described earlier, and a EcoRI site on the 5' end, and a NheI site on the 3' end were introduced, and subcloned into EcoRI and XbaI sites of pFLAG-CMV2-YFPN. For constructing BiFC expression vectors encoding YFPN- and YFPC-fused to the C-terminus of B55β, B55β2, B55β2mut, and B55βαβ, cDNAs of B55β, B55β2, B55β2mut, and B55βαβ were amplified by PCR using plasmids described earlier, and a HindIII site on the 5' end, and a XbaI site on the 3' end were introduced, and subcloned into HindIII and XbaI sites of pFLAG-CMV2-YFPN. For constructing BiFC expression vectors encoding YFPN- and YFPC-fused to the C-terminus of α4 or α4mut, cDNAs of α4 or α4mut were amplified by PCR using plasmids described earlier, an ApaI site on the 5' end, and a KpnI site on the 3' end were introduced, and subcloned into ApaI and KpnI sites of pCMV2-HA-YFPC. For CFP-fusion expression vector of Cα, cDNAs of Cα amplified by PCR using plasmid described earlier, a Flag-epitope tag and a BamHI site on the 5' end, and a EcoRI site on the 3' end were introduced, and subcloned into BamHI and EcoRI sites of pECFP-N1 (Clontech). For CFP-fusion expression vectors of B55β2 and B55β2mut, cDNAs of B55β2 and B55β2mut were amplified by PCR using plasmid described earlier, a Flag-epitope tag and a HindIII site on the 5' end, and a SalI site on the 3'end were introduced, and were subcloned into HindIII and SalI sites of pECFP-C1 (Clontech).
